# A Sweet Galactose Transfer: Metabolic Oligosaccharide Engineering as a Tool To Study Glycans in *Plasmodium* Infection

**DOI:** 10.1002/cbic.202000226

**Published:** 2020-05-14

**Authors:** Annabel Kitowski, Gonçalo J. L. Bernardes

**Affiliations:** ^1^ Instituto de Medicina Molecular João Lobo Antunes Faculdade de Medicina Universidade de Lisboa Avenida Professor Egas Moniz 1649-028 Lisboa Portugal; ^2^ Department of Chemistry University of Cambridge Lensfield Road Cambridge CB2 1EW UK

**Keywords:** bioorthogonal chemistry, carbohydrates, GLUT1 transporters, inverse electron-demand Diels-Alder, malaria

## Abstract

The introduction of chemical reporter groups into glycan structures through metabolic oligosaccharide engineering (MOE) followed by bio‐orthogonal ligation is an important tool to study glycosylation. We show the incorporation of synthetic galactose derivatives that bear terminal alkene groups in hepatic cells, with and without infection by *Plasmodium berghei* parasites, the causative agent of malaria. Additionally, we demonstrated the contribution of GLUT1 to the transport of these galactose derivatives, and observed a consistent increase in the uptake of these compounds going from naïve to *P. berghei*‐infected cells. Finally, we used MOE to study the interplay between *Plasmodium* parasites and their mosquito hosts, to reveal a possible transfer of galactose building blocks from the latter to the former. This strategy has the potential to provide new insights into *Plasmodium* glycobiology as well as for the identification and characterization of key glycan structures for further vaccine development.

## Graphical abstract

***Plasmodium*** **glycobiology**: The use of artificial galactose derivatives and labelling through iEDDA reaction in the liver stage of *Plasmodium* infection showed increased uptake in infected hepatic cells, through participation of GLUT1 transporters. Furthermore, unprotected derivatives are transferred from the mosquito host to the *Plasmodium berghei* parasite. This strategy has the potential to provide new insights into *Plasmodium* glycobiology.

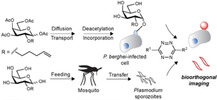

Metabolic oligosaccharide engineering (MOE) is an important tool in carbohydrate research and chemical biology.^1^ Next to proteins and nucleic acids, carbohydrates represent one of the largest groups of biomolecules, with a wide range of functionalities in health and disease states.^2^ Although, for example, changes in protein structures can be addressed through the corresponding RNA sequence, carbohydrate structures are not template‐encoded, which can complicate their investigation.^3^ With the introduction of chemical reporter groups in glycan structures through MOE, new strategies have been developed to investigate glycan structures in different disease settings. Over the years, different synthetic monosaccharide derivatives of *N*‐acetylmannosamine (ManNAc), *N*‐acetylglucosamine (GlcNAc), sialic acid and *N*‐acetylglucosamine (GalNAc) have been developed and used for MOE in different organisms and experimental settings.^4^ Typically MOE strategies use azides, alkynes, terminal or strained alkenes, diazo groups or nitrones as chemical reporter groups.2c, 4b Recent work has focussed on optimizing the required bioorthogonal labelling reactions in terms of reaction kinetics and selectivity. Initially the focus was on labelling reactions on the cell‐surface, but more recent studies showed intracellular labelling and tissue‐specific enrichment experiments.^5^

We decided to focus on the use of galactose, due to its apparent importance in the context of infection by *Plasmodium* parasites, the causative agents of malaria.^6^ The glycobiology of *Plasmodium* parasites has been amply discussed and is the subject of several studies aimed at the identification of new drug targets or vaccine antigens.^7^ Recent research suggests that the trisaccharide α‐Gal and corresponding anti‐α‐Gal antibodies play an important role in the acquisition of immunity against malaria.^8^ However, external factors are involved in the level of expression of these antibodies and it remains unclear how α‐Gal is formed on the parasite.^8^ During the course of a *Plasmodium* infection, sporozoites injected by an infected mosquito, travel in the blood stream from the initial injection site to the liver and invade hepatocytes. In fact, by means of a remarkable intrahepatic replication process, up to 40 000 merozoites are released from each infected hepatocyte, which initiates the symptomatic blood stage of infection.^9^ This important feature of the liver stage of *Plasmodium* infection makes it a major target for the development of therapies and vaccines. It has been shown that *Plasmodium* sporozoite proteins, like the circumsporozoite protein (CSP), are presented by infected hepatocytes during the liver stage and contribute, together with so far unidentified antigen structures, to the activation of CD8^+^ T cells.^10^ This observation, together with recent findings on the immunogenic properties of the α‐Gal epitope, suggested the potential presence of galactose containing glycan structures on infected cells. To test this hypothesis, we decided to use synthetic galactose derivatives in combination with MOE to investigate different stages of *Plasmodium* infection.8a

Baskin and co‐workers have just disclosed 6‐alkynyl uridine diphosphate galactose derivatives, followed by click chemistry tagging, to visualize glycosylation during zebrafish development. Together with the work presented here, it constitutes the first examples of the use of galactose reporters with MOE to study the roles of galactose in different biological settings.^11^

Starting from commercially available 1,6‐anhydro‐3,4‐isopropylidene‐β‐d‐galactopyranose, three galactose derivatives with differently sized terminal‐alkene reporter groups were synthesized (Figure 1a). The introduced chemical reporters were addressed after metabolic incorporation through inverse electron‐demand Diels‐Alder (iEDDA) reaction with 6‐methyl‐tetrazine compounds. Briefly, derivatives **2 a**, **2 b** and **5** were obtained through classical Williamson‐ether synthesis, followed by deprotection and acetylation of the monosaccharide. Zémplen deacetylation resulted in deprotected derivatives **3 a**, **3 b** and **6** (see the Supporting Information). The determination of the second‐order rate constants for these derivatives in the iEDDA reaction was conducted in 96‐well‐plates with a microplate reader, and the decrease in specific tetrazine absorbance at 530 nm was monitored. The measurements were performed at 37 °c in PBS, pH 7.4. After determining the pseudo‐first‐order rate constant *k*_obs_ at different concentrations, an exponential decay function was used to calculate the corresponding second‐order rate constants (Figure S1 in the Supporting Information). In agreement with previous studies that used mannosamine derivatives, the longer pentenyl‐substituted derivatives showed faster kinetics when reacted with 6‐methyl‐tetrazine‐amine relative to the shorter allyl‐substituted derivative (Figure 1b).^12^

**Figure 1:**
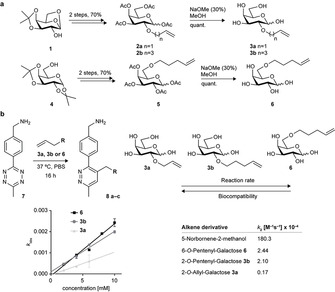
a) Synthesis of galactose derivatives **2 a**, **2 b**, **3 a**, **3 b**, **5** and **6** for metabolic labelling. b) Kinetic measurements and determination of the second‐order rate constant for the iEDDA reaction between tetrazine **7** and monosaccharides **3 a**, **3 b** and **6**.

We started by employing these galactose derivatives to study the liver stage of a *Plasmodium* infection. We considered the importance of the α‐Gal epitope for anti‐malarial immunity and sought to understand whether a change in galactose‐containing glycans was observed on hepatocytes after infection with the *Plasmodium* parasite.8b We started by investigating the metabolic incorporation of the synthesized galactose derivatives into human hepatoma cells. In general, cells were grown for 48–72 h in the presence of 100 μM of **2 a**, **2 b**, **5** or natural counterpart penta‐*O*‐acetyl‐d‐galactose **9**, before treatment with 6‐methyl‐tetrazine‐PEG4‐biotin, which served as a handle for further labelling (Figure 2a). All derivatives were nontoxic at the concentrations employed in the experiments (Figure S2). Huh7 and HepG2 cells were grown for 72 h in complete Dulbecco's modified Eagle's medium, supplemented with 100 μM of the galactose derivatives **2 a**, **2 b**, **5** and control **9**, followed by labelling of the cell membrane with 6‐methyl‐tetrazine‐PEG4‐biotin, which served as an handle for further labelling, and Alexa‐Fluor‐568‐streptavidin (Figure 2a). Analysis and quantification of the fluorescence intensity was performed by using confocal point‐scanning microscopy and ImageJ software. In compliance with the kinetic evaluations, a stronger fluorescence signal was detected for derivatives **2 b** and **5** relative to **2 a** (Figures 2b and S3). The marginal signal increase observed for derivative **5** relative to **2 b** may be explained by the slightly higher kinetics of **5**, due to its lower steric hindrance, as a result of the presence of the pentenyl group in C6 instead of C2. Compounds **5** and **2 b** represent unnatural galactose derivatives, so we do not want to exclude different interactions of these molecules with enzymes involved in glycan biosynthesis. The switch of the terminal alkene reporter group from C2 to C6 position can influence the recognition process by necessary enzymes and therefore change the incorporation rate into glycan structures. Epimerization to glucose was excluded because no labelling was observed after treatment with β‐galactosidase (Figure 2c). The incorporation of **2 b** or **5** into glycoprotein structures was also corroborated in pull‐down experiments (Figure S11). Additionally, inhibition experiments with **9** and benzyl 2‐acetamido‐2‐deoxy‐d‐galactopyranoside confirmed the incorporation of **2 b** also in *O*‐glycans in addition to N‐glycans (Figure S12 and S13).

**Figure 2:**
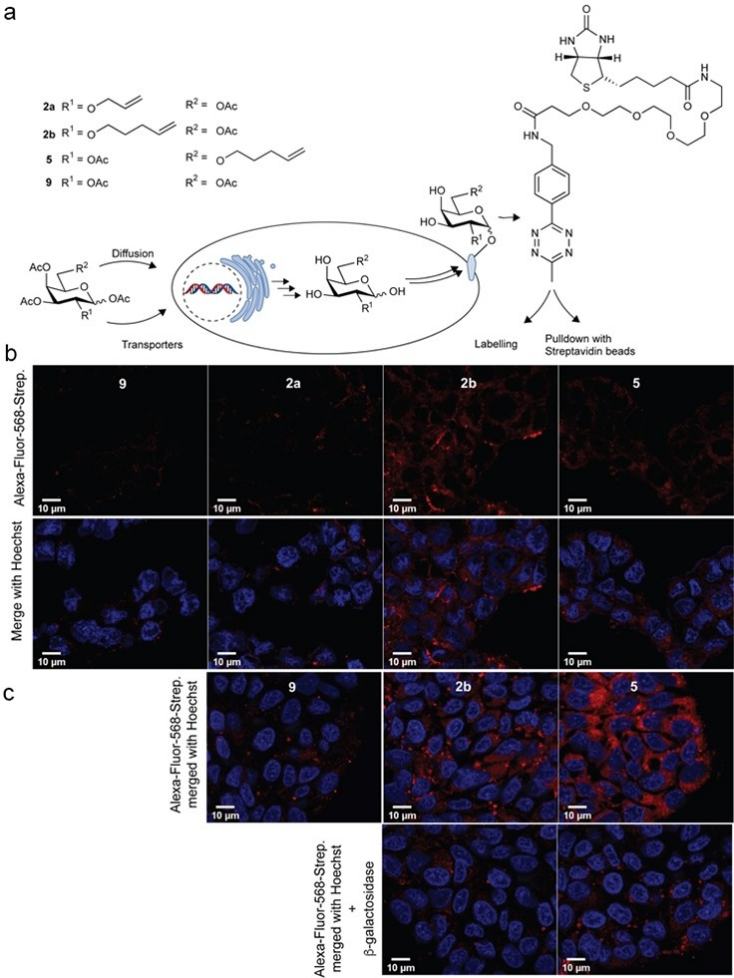
a) Schematic description of the incorporation of galactose derivatives into cell‐surface glycans and targeting strategies with 6‐methyl‐tetrazine‐PEG4‐biotin. b) Incorporation of **2 a**, **2 b**, and **5** in Huh7 cells, labelling with Alexa‐Fluor‐568‐streptavidin (red) and Hoechst (blue). c) Incorporation of **2 b, 5** and **9** into HepG2 cells and treatment with β‐galactosidase (1 U, 30 min, 37 °C), labelling with Alexa‐Fluor‐568‐streptavidin (red) and Hoechst (blue). Scale bars: 10 μm.

Chen and co‐workers reported cysteine S‐glycosylation by acetylated unnatural monosaccharides during MOE.^13^ Thus, we decided to attempt the direct incorporation of non‐acetylated derivative **3 b** in glycans present in the cell membrane of HepG2 cells (Figure S5). Growing HepG2 cells for 72 h in the presence of **3 b** (100 μM) resulted in the successful incorporation of the unprotected galactose derivative, which could be fluorescently labelled by using our iEDDA approach. Our data suggests the incorporation of the galactose derivatives into the newly synthetized glycan structures rather than S‐glycosylation.

Having shown successful incorporation of the artificial galactose derivatives by human hepatic cells, we then sought to investigate how this incorporation might change in the context of the liver stage of *Plasmodium* infection. To this end, HepG2 cells were infected with GFP‐expressing *P. berghei* sporozoites freshly isolated from salivary glands of infected *Anopheles* mosquitoes, and the cells were grown in the presence of 100 μM of **2 b** or **9** until 48 h post‐infection (hpi). Galactose incorporation was analysed by confocal point‐scanning microscopy and imaging flow cytometry (Figure 3a). Confocal point‐scanning microscopy showed a clear labelling of the glycans in the cell membrane, both in uninfected and in *P. berghei*‐infected cells (Figure 3b). Incorporation of **2 b** was then analysed by imaging flow cytometry 48 h after sporozoite addition, which allows us to distinguish between infected cells, which contain the GFP‐expressing parasite, and uninfected cells (Figure S6). To answer our question regarding the change of galactose‐containing glycans in *P. berghei*‐infected hepatocytes, we performed our MOE strategy using iEDDA reaction with 6‐methyl‐tetrazine‐PEG4‐biotin and subsequent labelling with Alexa‐Fluor‐568‐streptavidin. Analysing this signal indicated a slight yet consistent difference in uptake and incorporation of **2 b** by hepatocytes that were infected by *P. berghei* sporozoites when compared to naïve hepatocytes (Figure 3c), although this effect was not statistically significant. Furthermore, a consistent difference in fluorescence was also detected between infected cells and neighbouring uninfected cells.

**Figure 3:**
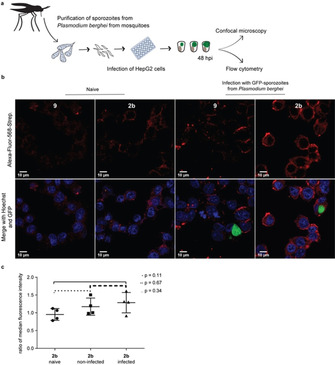
a) Workflow for the infection of HepG2 cells with sporozoites from *P. berghei* and metabolic incorporation of **2 b**. b) Metabolic labelling of HepG2 cells with **2 b** or **9** after infection with GFP‐sporozoites from *P. berghei* (green), labelling with Alexa‐Fluor‐568‐streptavidin (red) and Hoechst (blue) 48 hpi. Scale bars: 10 μm. c) Ratio of median fluorescence intensity from Alexa‐Fluor‐568‐streptavidin of naïve, uninfected and infected HepG2 cells, acquired in Amnis ImageStreamX, ratio against corresponding control with **9**. Data from four individual experiments were pooled, each data point represents the median fluorescence intensity of 80 (infected) or 2000 cells (naïve, uninfected), two‐tailed Mann‐Whitney.

The observation of a moderate increase of incorporated galactose derivative **2 b**, could suggest a change in certain metabolic events in *P. berghei*‐infected hepatocytes. We started to look into metabolic hallmarks, which are known to occur during the course of parasite development in the infected hepatic cell. Specifically, the importance of hexose transporters for processes related to the energy metabolism of the developing parasite caught our attention. It has been shown that, during the blood stage of *Plasmodium* infection, glucose is transported through GLUT1 into erythrocytes and taken up by the parasite by a facilitative hexose transporter (PfHT).^14^ GLUT1 is also expressed in liver cells and it has been shown that the enhanced translocation of this transporter to the membrane of *P. berghei*‐infected hepatic cells, leads to increased glucose uptake during the later stages of *Plasmodium* liver infection.^15^ GLUT1 belongs to a family of class I facilitative glucose transporters, which also facilitates the diffusion of galactose.^16^ We hypothesized that the coherent increase observed in the uptake of galactose derivative **2 b** by infected cells over naïve ones, could be related with the reported increased translocation of GLUT1 to the cell membrane of those cells. In this case, our artificial galactose derivative **2 b** would also be transported through the GLUT1 transporter.

To test this hypothesis, we selected two specific GLUT1 inhibitors, WZB117 **10** and STF31 **11**, and nonspecific inhibitor cytochalasin B **12** to block this transporter during the period of incubation with galactose derivative **2 b** (Figure 4a). Briefly, HepG2 cells were grown as before in the presence of **2 b** (100 μM) for 72 h, in the presence of 10 μM of each inhibitor **10**, **11** or **12**. Interestingly, a decrease in fluorescence intensity was observed in the presence of all the inhibitors employed, and a dose‐dependent decline in fluorescence intensity in the presence of the selective inhibitor **10** (Figures 4b, c and S8). These results confirm the ability of GLUT1 to transport artificial galactose structures into the cytosol. These results further support the notion that *P. berghei*‐infected hepatic cells have a higher influx of derivative **2 b**, due to the increased translocation of GLUT1 transporters to the cell membrane of these cells.

**Figure 4:**
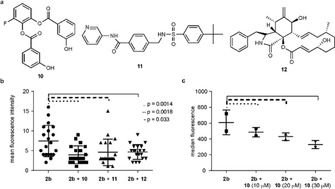
a) GLUT1 inhibitors WZB117 **10**, STF31 **11** and cytochalasine B **12**. b) Mean fluorescence intensity after metabolic incorporation of **2 b** in the presence of 10 μM of inhibitors **10**, **11** and **12**; two‐tailed Mann‐Whitney. c) Concentration‐dependent decrease in fluorescence intensity after metabolic incorporation of **2 b** with increasing concentration of inhibitor **10**, pool of two independent experiments; two‐tailed Mann‐Whitney.

Having employed artificial galactose derivatives to investigate galactose uptake by *Plasmodium*‐infected hepatic cells, we then sought to extend our MOE approach to the study of the *Plasmodium* parasite itself. As previously mentioned, the presence of IgM antibodies against the trisaccharide α‐Gal was reported to provide a protective effect against malaria transmission.8a Although the α‐Gal epitope has been detected on sporozoites from *P. falciparum*, *P. berghei* and *P. yoelii*, the enzymes required for assembly of this epitope have not yet been identified.7a, 8a Residual levels of this epitope were also detected in proteins from salivary glands of non‐infected *Anopheles* mosquitoes, which could suggest a transfer of substrates or structures from the mosquito host to the parasite.8a We therefore employed our MOE strategy to test this hypothesis and assess a possible transfer of administered galactose compounds from *Anopheles* mosquitoes to *Plasmodium* parasites. To this end, female *A. stephensi* mosquitoes were fed with a sugar solution, supplemented with either **2 b** or its deprotected counterpart **3 b**. Pentaacetylated galactose **9** or galactose **13** were used as negative controls in these experiments. The feeding was initiated after the mosquitoes’ infectious blood meal and was maintained for 21–24 days. After this period, mosquito salivary glands were collected and *P. berghei* sporozoites were isolated and analysed by imaging flow cytometry (Figure 5a). Our results show that the fluorescence intensity, which corresponds to the incorporated galactose derivatives **2 b** or **3 b**, was significantly higher in sporozoites dissected from mosquitoes fed on deprotected galactose derivative **3 b**, than that observed in sporozoites obtained from control mosquitoes. Thus, although the acetylated galactose derivative **2 b** seemed not to be transferred from the mosquito to the parasite, our labelling approach by means of iEDDA reaction indicated the transfer of derivative **3 b** (Figure 5b). This observed transfer of galactose molecules from the mosquito host to the *Plasmodium* parasite could explain the reported detections of α‐Gal epitopes on the parasite surface. Interestingly, the sporozoites obtained from mosquitoes fed on derivative **3 b**, seem to be slightly bigger in size than sporozoites obtained from the control mosquitoes, whereas a smaller number of parasites was observed in the former than in the latter (Figures S9 and S10). Further investigations into the origin and biosynthetic pathways of the α‐Gal epitope on *Plasmodium* parasites would require many more factors to be taken into account. Metabolic labelling experiments with the isolated parasite or in different developmental stages can shed light on this question, however, this should be addressed in a separated work.

**Figure 5:**
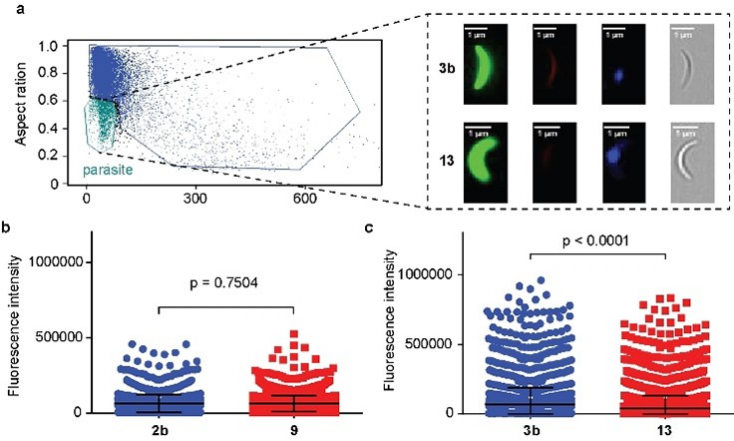
a) Gating strategy for acquisition of *Plasmodium berghei* sporozoites after incorporation of **3 b**. Scale bars: 1 μm. b) Comparison of fluorescence intensity after labelling of sporozoites with 6‐methyl‐tetrazine‐sulfo‐Cy3, after incorporation of galactose derivative **2 b** or control **9**; two‐tailed Mann‐Whitney. c) Comparison of fluorescence intensity after labelling of sporozoites with 6‐methyl‐tetrazine‐sulfo‐Cy3, after incorporation of galactose derivative **3 b** or control **13**; two‐tailed Mann‐Whitney.

This proof‐of‐concept study demonstrates the application of MOE with artificial galactose derivatives in the context of *Plasmodium* infection. We showed the participation of GLUT1 transporters in the facilitated diffusion of the artificial galactose derivatives and a reliable enhancement in the amount of galactose derivative incorporated in *P. berghei*‐infected hepatic cells relative to that observed in naïve cells. The ability of hexose transporters like GLUT1 to transport unnatural monosaccharide derivatives can be an important tool for further studies in this area. Screening of different unnatural monosaccharide compounds could identify candidates, which are significantly increased in *P. berghei*‐infected cells and which could be used as targeting agents, for example in drug‐conjugates. The application of per‐acetylated monosaccharide derivatives for MOE experiments is built on the assumption of an endocytic or diffusion‐based cell entry of these compounds. Gilormini et al. demonstrated the participation of an as yet unknown transporter in the translocation of unnatural mannose derivatives, which is supported by earlier studies by Varki and co‐workers.^17^ In this context, our observation of a facilitated diffusion of presented galactose derivative **2 b** through GLUT1 contributes to the notion of a more complex scenario then simple diffusion, which should not be excluded as a parallel transport route. In our study, the participation of GLUT1 provides a reasonable explanation for our observations. For future applications of MOE, such transporters could provide a tool for, for example enrichment of unnatural sugars in a specific tissue or cell organelle.

We further employed the MOE approach to demonstrate the transfer of the artificial galactose derivative **3 b** from the mosquito host to the parasite. The impact of the introduction of an artificial sugar structure on the quality and infectivity of the sporozoites requires further investigation. Although we have not addressed yet possible sugar transfers in other life‐stages of the parasite, these experiments could be used to gain further understand of the glycobiology processes of the *Plasmodium* parasite. In summary, the approach presented in this study demonstrates the usefulness of MOE for further investigations on the *Plasmodium* glycobiology and might open new possibilities for the identification and characterization of important glycan structures for vaccine development.

## Conflict of interest

The authors declare no conflict of interest.

## Supporting information

As a service to our authors and readers, this journal provides supporting information supplied by the authors. Such materials are peer reviewed and may be re‐organized for online delivery, but are not copy‐edited or typeset. Technical support issues arising from supporting information (other than missing files) should be addressed to the authors.

Supplementary
