# Supplementary material for: A Sweet Galactose Transfer: Metabolic Oligosaccharide Engineering as a Tool To Study Glycans in Plasmodium Infection
Source: Chembiochem. 2020 May 14;21(18):2696–700. doi: 10.1002/cbic.202000226 (PMC7540713; doi:10.1002/cbic.202000226)
Supplement: Supplementary file 1 — Supplementary [file CBIC-21-2696-s001.pdf]

ChemBioChem

Supporting Information

A Sweet Galactose Transfer: Metabolic Oligosaccharide Engineering as a Tool To Study Glycans in *Plasmodium* Infection

Annabel Kitowski and Gonçalo J. L. Bernardes*© 2020 The Authors. Published by Wiley-VCH Verlag GmbH & Co. KGaA. This is an open access article under the terms of the Creative Commons Attribution License, which permits use, distribution and reproduction in any medium, provided the original work is properly cited.

Supporting Information

Table of Contents

1. Supporting Figures ... 2
2. General Procedures ... 14
   2.1. Chemical Synthesis ... 14
      2.1.1. Synthesis of 2-*O*-allyl-1,3,4,6-tetra-*O*-acetly-galactopyranose (**2a**) ... 14
      2.1.2. Synthesis of 2-*O*-allyl-galactopyranose (**3a**) ... 16
      2.1.3. Synthesis of 2-*O*-pentenyl-1,3,4,6-tetra-*O*-acetyl-galactopyranose (**2b**) ... 17
      2.1.4. Synthesis of 2-*O*-pentenyl-galactopyranose (**3b**) ... 18
      2.1.5. Synthesis of 6-*O*-pentenyl-1,2,3,4-tetra-*O*-acetyl-galactopyranose (**5**) ... 19
      2.1.6. Synthesis of 6-*O*-pentenyl-galactopyranose (**6**) ... 21
   2.2. Kinetic Studies ... 22
   2.3. Cell Culture ... 22
      2.3.1. Cell toxicity ... 23
      2.3.2. Metabolic Labeling in Huh7 and HepG2 cells ... 23
      2.3.3. Metabolic labeling of HepG2 cells in presence of inhibitors for GLUT1 ... 24
      2.3.4. Metabolic labeling of HepG2 cells for cell lysis and pull-down ... 25
      2.3.5. Competition experiment and Inhibition of *O*-glycosylation ... 26
      2.3.6. Release of cell surface glycans ... 27
   2.4. Infection Studies ... 27
      2.4.1. Infection of HepG2 cells and analysis by confocal microscopy ... 28
      2.4.2. Infection of HepG2 cells and analysis by flow cytometry and imaging flow cytometry ... 28
   3. NMR Spectra ... 30

1. Supporting Figures

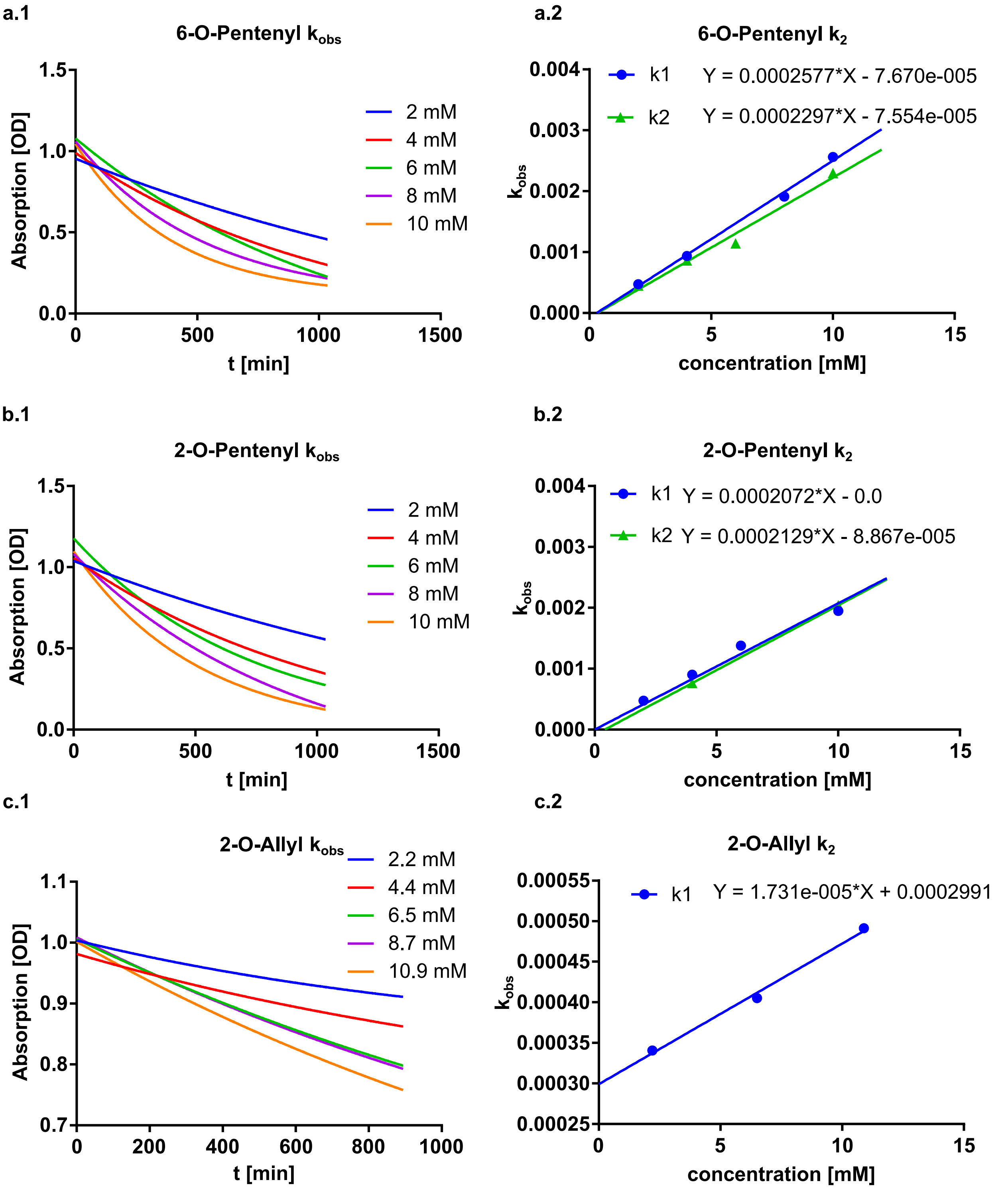

Figure S1: **a.1.** Decrease in absorbance of absorbance of 6-Methyl-tetrazine-amine at 530 nm with increasing concentrations of 6-O-Pentenyl-galactose in PBS pH = 7.4 at 37 °C. **a.2.** Plot of k_{obs} vs. the corresponding concentration of 6-O-Pentenyl-galactose **b.1.** Decrease in absorbance of absorbance of 6-Methyl-tetrazine-amine at 530 nm with increasing concentrations of 2-O-Pentenyl-galactose in PBS pH = 7.4 at 37 °C. **b.2.** Plot of k_{obs} vs. the corresponding concentration of 2-O-Pentenyl-galactose **c.1.**

Decrease in absorbance of absorbance of 6-Methyl-tetrazine-amine at 530 nm with increasing concentrations of 2-O-Allyl-galactose in PBS pH = 7.4 at 37 °C. **c.2.** Plot of k_{obs} vs. the corresponding concentration of 2-O-Allyl-galactose.

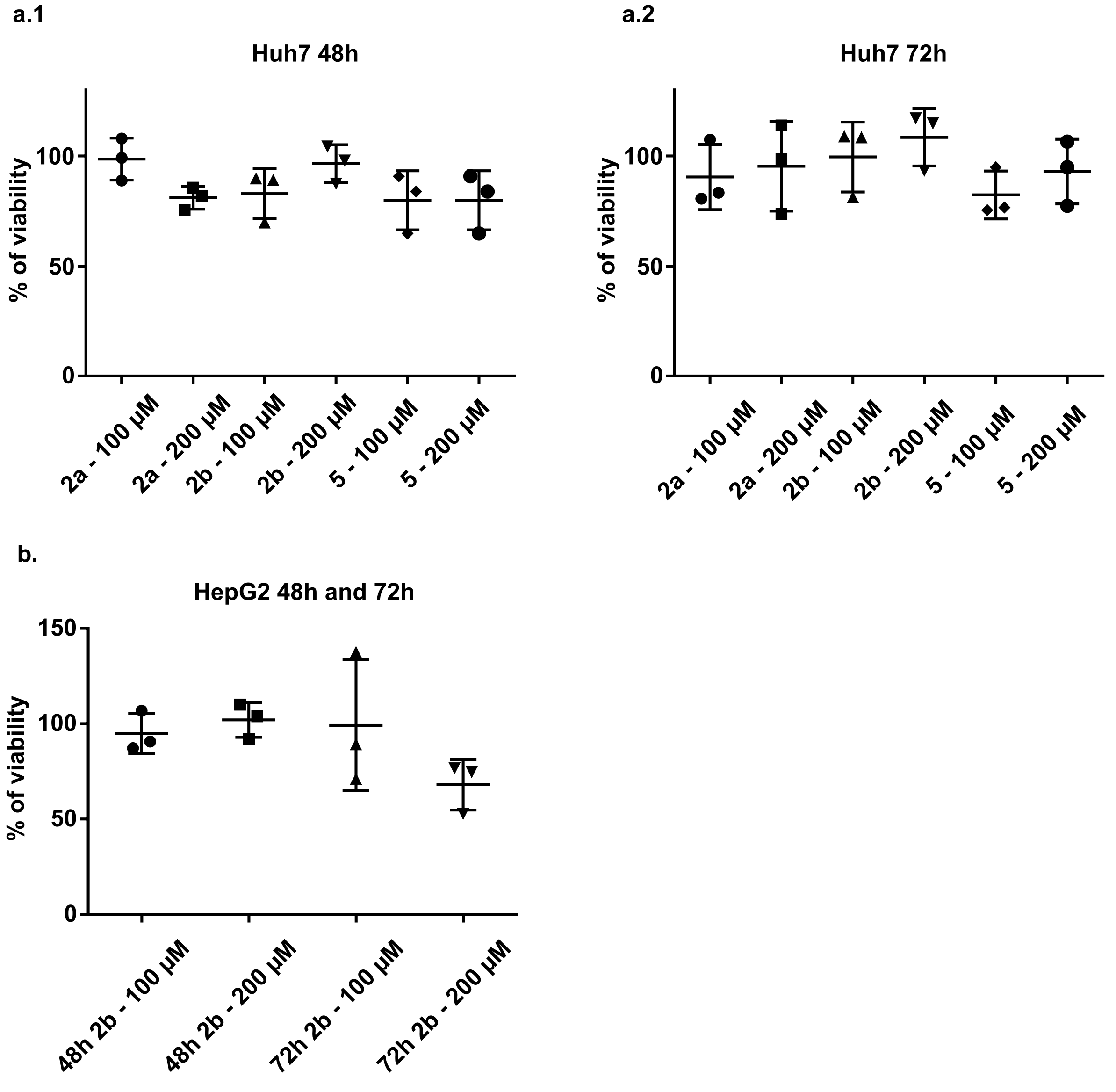

Figure S2: **a.1.** Viability of Huh7 cells incubated with artificial galactose derivatives for 48h, illustrated as percentage of maximum **a.2.** Viability of Huh7 cells incubated with artificial galactose derivatives for 72h, illustrated as percentage of maximum **b.** Viability of HepG2 cells incubated with artificial galactose derivatives for 48h and 72h, illustrated as percentage of maximum. Combined data from three independent experiments.

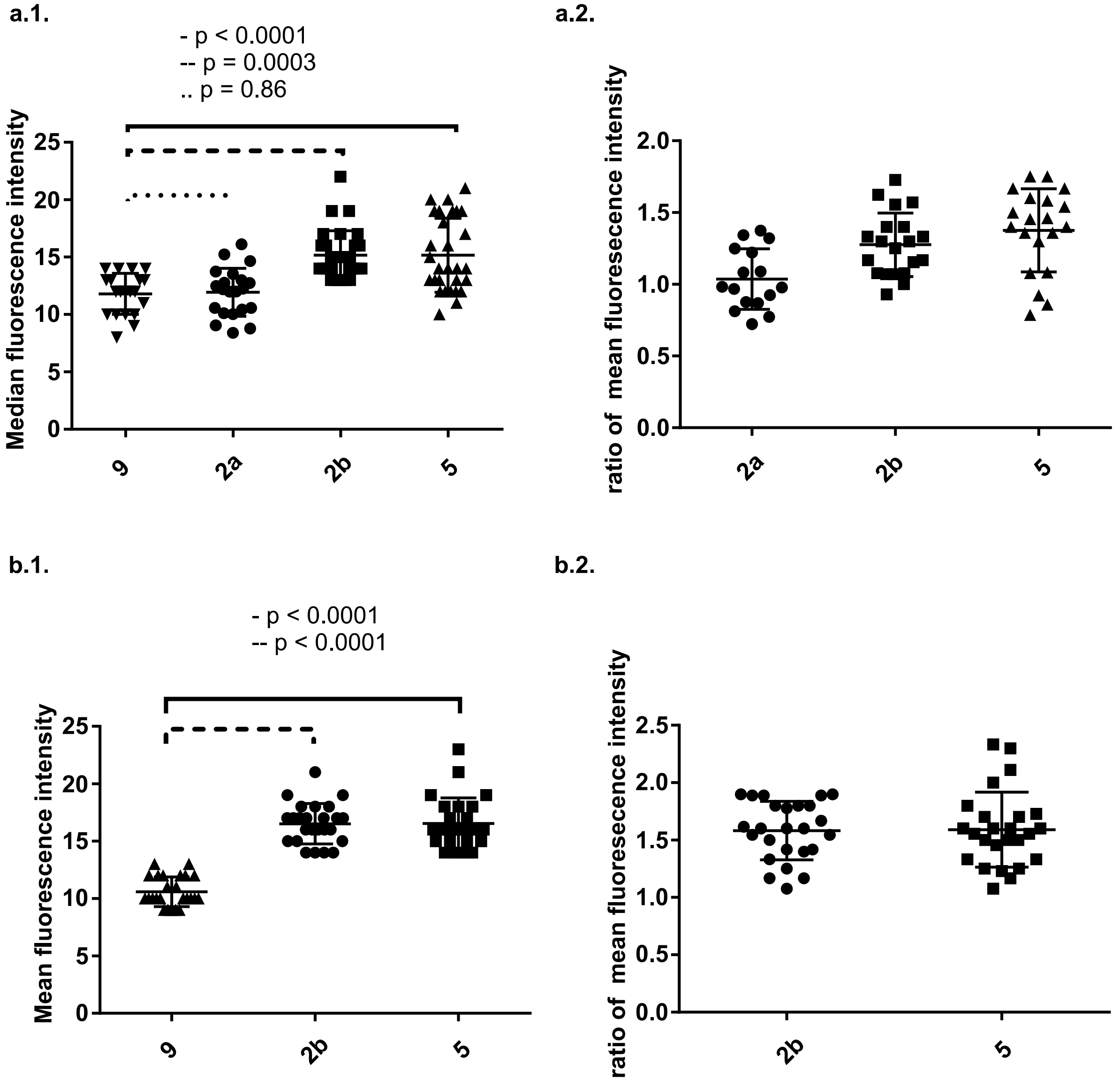

Figure S3: **a.1.** Quantification of Mean fluorescence intensity by confocal point-scanning micoroscopy after metabolic incorporation of **2a**, **2b** and **5** into cell membrane glycans of Huh7 cells, **9** was used as negative control. **a.2.** Ratio of mean fluorescence intensity against control **9** after metabolic incorporation of **2a**, **2b** and **5** in Huh7 cells. **b.2.** Quantification of Mean fluorescence intensity by confocal point-scanning microscopy after metabolic incorporation of **2b** and **5** into cell membrane glycans of HepG2 cells, **9** was used as a negative control. **b.2.** Ratio of mean fluorescence intensity against control **9** after metabolic incorporation of **2b** and **5** in HepG2 cells. Representative data from one out of three experiments, analysis of five to six picture per condition and 5-6 cells per picture, Two-tailed Mann-Whitney for non-parametric distribution, D'Agostino and Pearson omnibus normality test was performed for each data set.

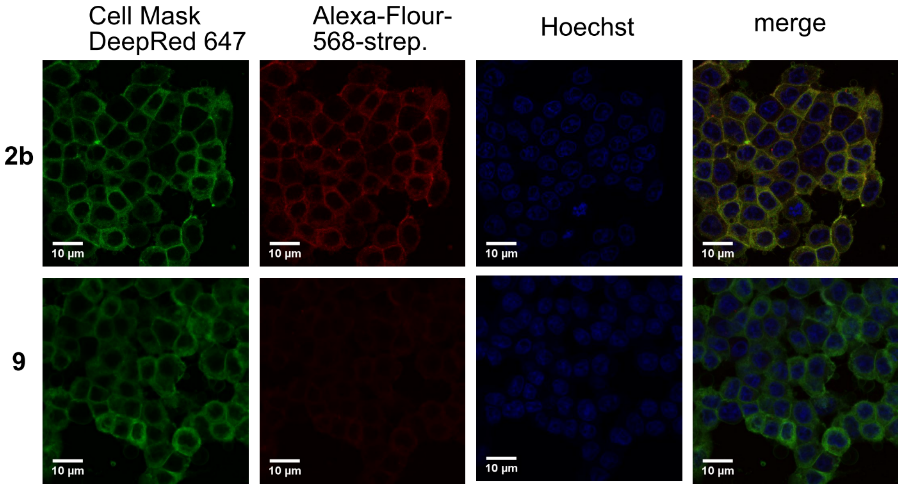

Figure S4: Metabolic incorporation of **2b** into HepG2 cells, labeling with 6-methyl-tetrazine-PEG4-biotin and Alexa-Flour-568-streptavidin (red). Co-staining of the cell membrane with CellMask Deep Red Plasma membrane stain (green). Nuclei were stained with Hoechst 33342, **9** was used as negative control.

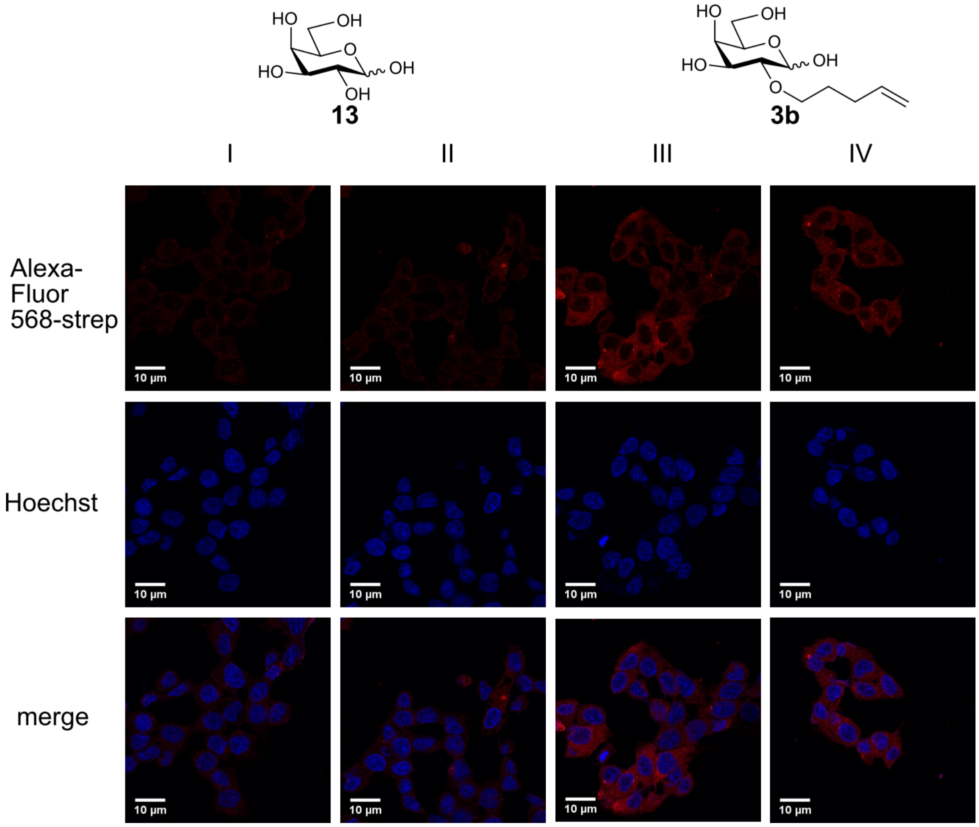

Figure S5: Incorporation of **13** and **3b** in HepG2 cells, labelling with Alexa-Fluor-568-streptavidin (red) and Hoechst (blue). I and III, as well as II and IV represent two independent experiments.

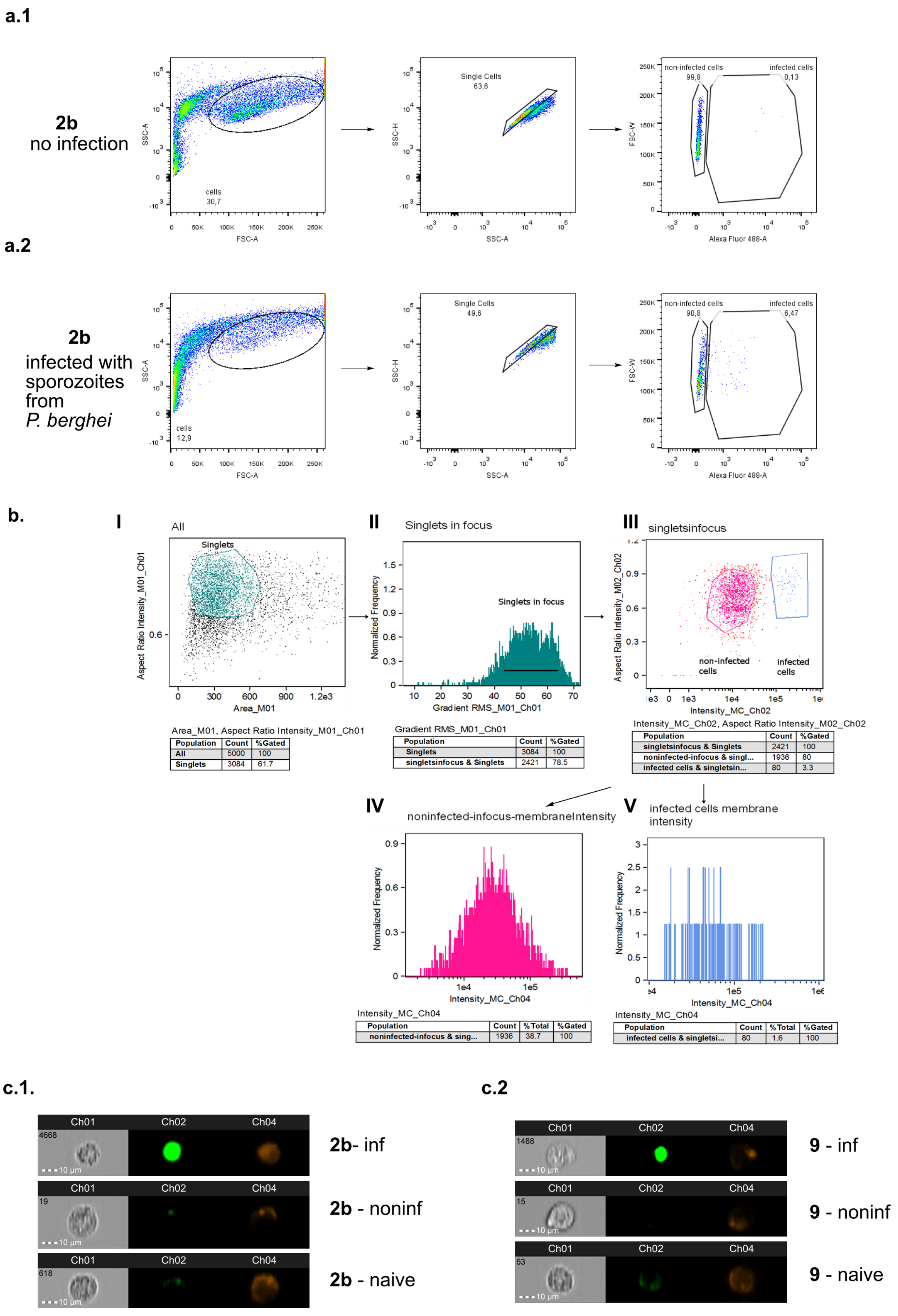

a.1
2b
no infection
cells
30,7
Single Cells
63,6
non-infected cells
99,8
infected cells
0,13
SSC-A
SSC-H
FSC-A
FSC-W
Alexa Fluor 488-A
a.2
2b
infected with sporozoites from *P. berghei*
cells
12,9
Single Cells
49,6
non-infected cells
90,8
infected cells
6,47
b.
I
All
Singlets
Aspect Ratio Intensity_M01_Ch01
Area_M01
Area_M01, Aspect Ratio Intensity_M01_Ch01
Population | Count | %Gated
All | 5000 | 100
Singlets | 3084 | 61.7
II
Singlets in focus
Normalized Frequency
Gradient RMS_M01_Ch01
Population | Count | %Gated
Singlets | 3084 | 100
singletsinfocus & Singlets | 2421 | 78.5
III
singletsinfocus
Aspect Ratio Intensity_M02_Ch02
non-infected cells
infected cells
Intensity_MC_Ch02
Intensity_MC_Ch02, Aspect Ratio Intensity_M02_Ch02
Population | Count | %Gated
singletsinfocus & Singlets | 2421 | 100
noninfected-infocus & singl... | 1936 | 80
infected cells & singletsin... | 80 | 3.3
IV
noninfected-infocus-membraneIntensity
Normalized Frequency
Intensity_MC_Ch04
Population | Count | %Total | %Gated
noninfected-infocus & sing... | 1936 | 38.7 | 100
V
infected cells membrane intensity
Normalized Frequency
Intensity_MC_Ch04
Population | Count | %Total | %Gated
infected cells & singletsi... | 80 | 1.6 | 100
c.1.
Ch01
Ch02
Ch04
10 µm
2b- inf
2b - noninf
2b - naive
c.2
9 - inf
9 - noninf
9 - naive

Figure S6: a.1/2. Gating strategy during the analysis of metabolic incorporation of **2b** in HepG2 cell membrane glycans by flow cytometry, selection of live cells, single cells and infected vs. non-infected cells. **b.** Gating strategy during the analysis of metabolic incorporation of **2b** in HepG2 cell membrane glycans by imaging flow cytometry, selection of single cells (I), single cells in focus (II), separation of infected and non-infected cells based on the intensity of GFP (Ch02, III), Fluorescence intensity resulting of the incorporation of **2b** in non-infected (IV) and infected cells (V). **c.1.** representative pictures acquired in Amnis ImageStreamX MarkII, HepG2 cells after metabolic incorporation of **2b**, infected, non_infected and naïve. **c.2.** representative pictures acquired in Amnis ImageStreamX MarkII, HepG2 cells after metabolic incorporation of contol **9**, infected, non-infected and naïve.

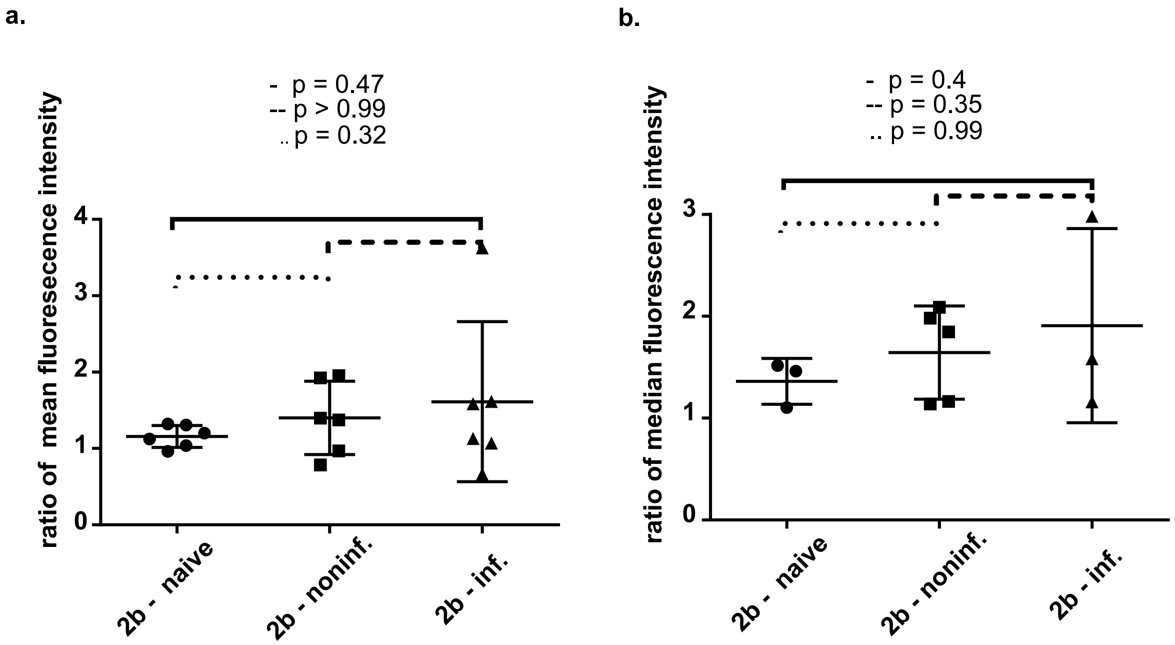

Figure S7: a. Quantification of fluorescence intensity resulting from incorporated galactose derivative **2b** by confocal point-scanning microscopy, Data representative from one experiment out of three, each data point represents the mean fluorescence intensity of all single cells within one picture, two-tailed Mann-Whitney test. **b.** Quantification of fluorescence intensity resulting from incorporated galactose derivative **2b** by flow cytometry, Combined data from three independent experiments, each data point represents the median intensity of 2000-3000 gated single cells, two-tailed Mann-Whitney test.

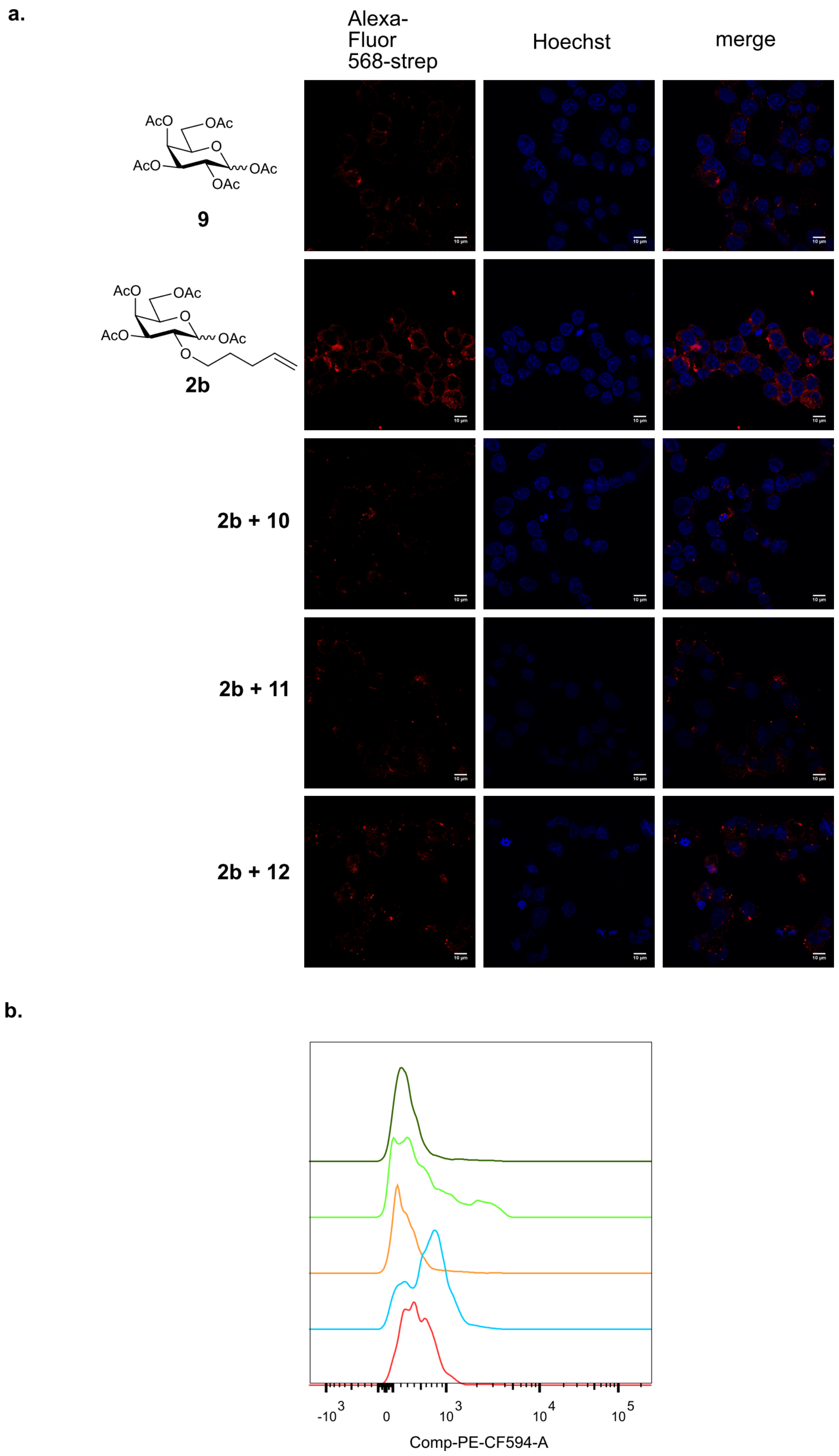
a.
Alexa-Fluor 568-strep
Hoechst
merge
AcO
OAc
O
AcO
OAc
OAc
9
2b
2b + 10
2b + 11
2b + 12
b.
-10^3
0
10^3
10^4
10^5
Comp-PE-CF594-A

Figure S8: a. Metabolic incorporation of **2b** into HepG2 cell membrane glycans, without and in presence of inhibitors **10-12**. Pentaacetyl galactose **9** was used as negative control. **b.** Half-offset histograms of fluorescence intensity in channel PE-CF594-A, normalized mode, after metabolic incorporation of derivative **2b** in HepG2 cells, without inhibitor (blue), with increasing concentrations of WZB117 **10**, 10 µM (orange), 20 µM (green), 30 µM (dark green). Pentaacetyl galactose **9** was used as negative control.

a.

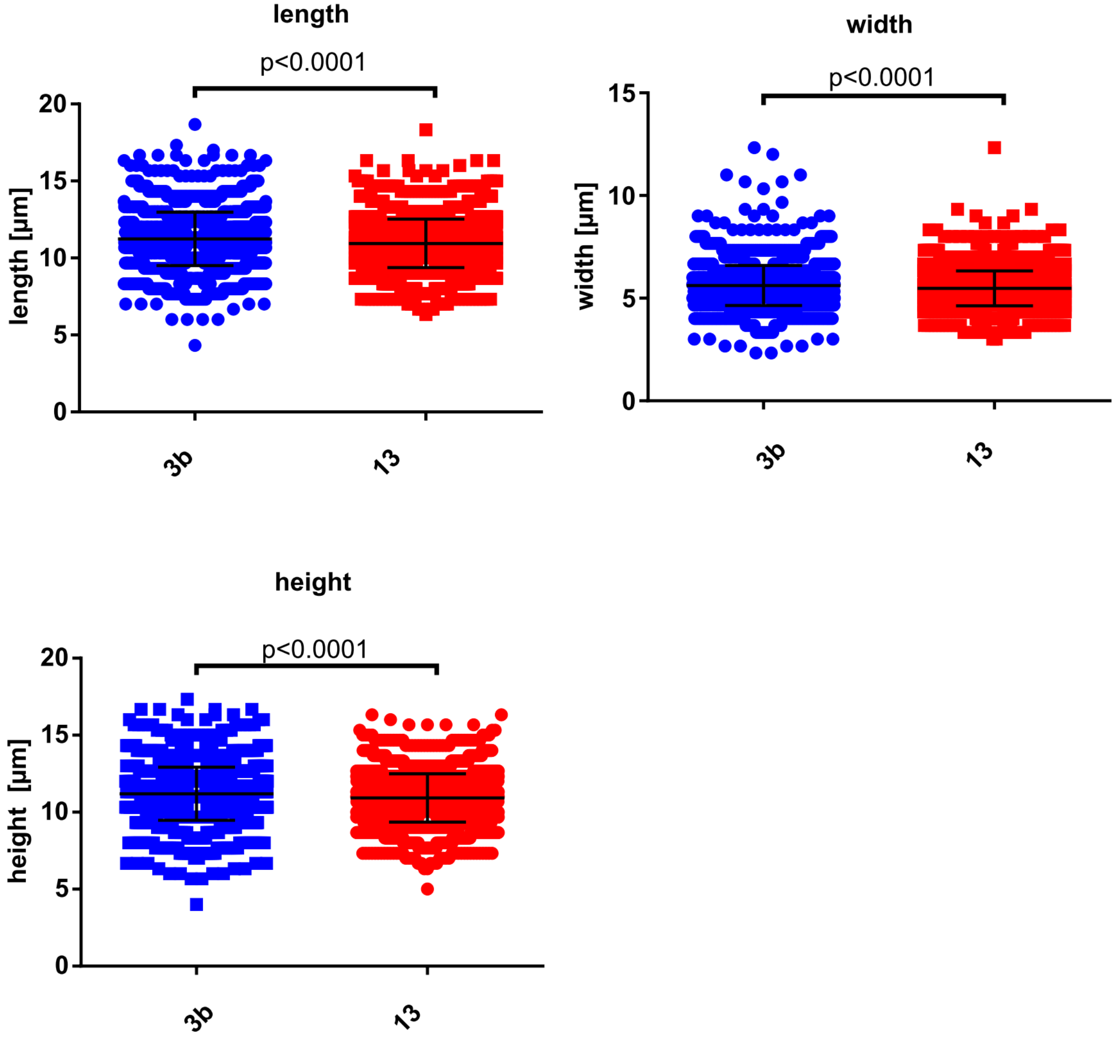
length
p<0.0001
length [µm]
20
15
10
5
0
3b
13
width
p<0.0001
width [µm]
15
10
5
0
3b
13
height
p<0.0001
height [µm]
20
15
10
5
0
3b
13

b.1.

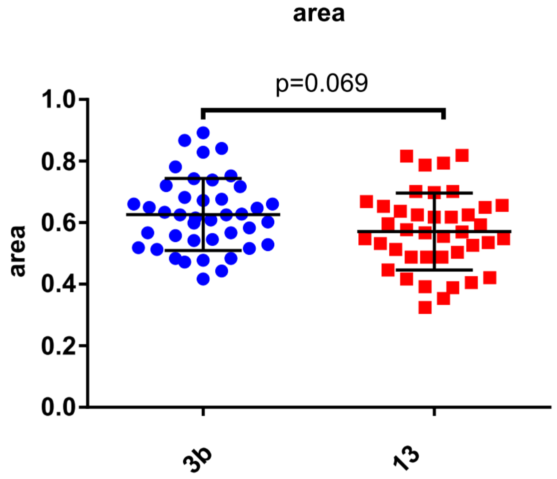
area
p=0.069
area
1.0
0.8
0.6
0.4
0.2
0.0
3b
13

b.2.

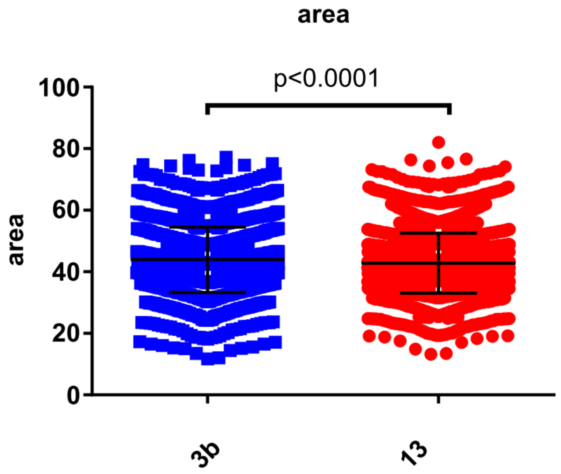
area
p<0.0001
area
100
80
60
40
20
0
3b
13

Figure S9: a. Shape parameters of *Plasmodium berghei* parasites after feeding with **3b** or control **13**, acquisition in Amnis ImageStreamX MarkII, two-tailed Mann-Whitney. **b.1.** Determination of the parasite size/area of representative pictures acquired with Amnis ImageStreamX MarkII, measurements were performed using ImageJ software package, n = 50, two-tailed Mann-Whitney. **b.2.** Determination of the parasite size/area of all acquired pictures with Amnis ImageStreamX MarkII, n=2000-3000, two-tailed Mann-Whitney.

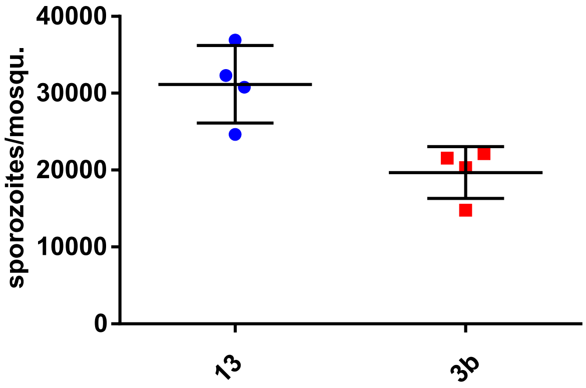

Figure S 10: Sporozoite number per mosquito, for mosquitoes receiving galactose derivative **3b** or control **13**.

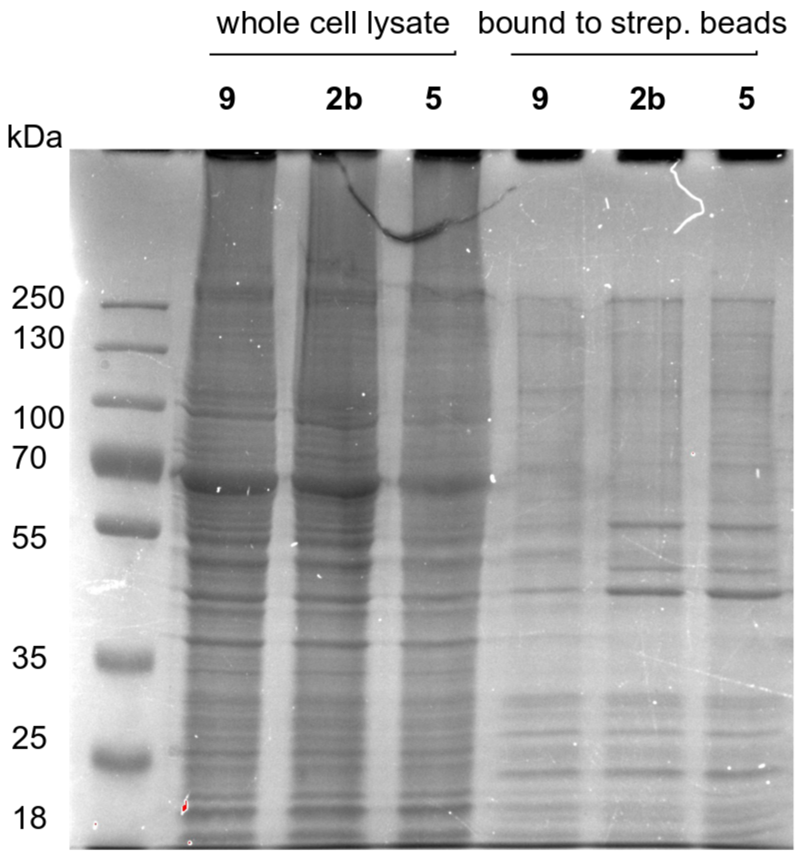

Figure S11: Coomassie blue staining of whole protein cell lysate and protein samples bound to streptavidin magnetic beads from HepG2 cells, cells were grown for 72h with 100 µM of **9**, **2b** or **5**.

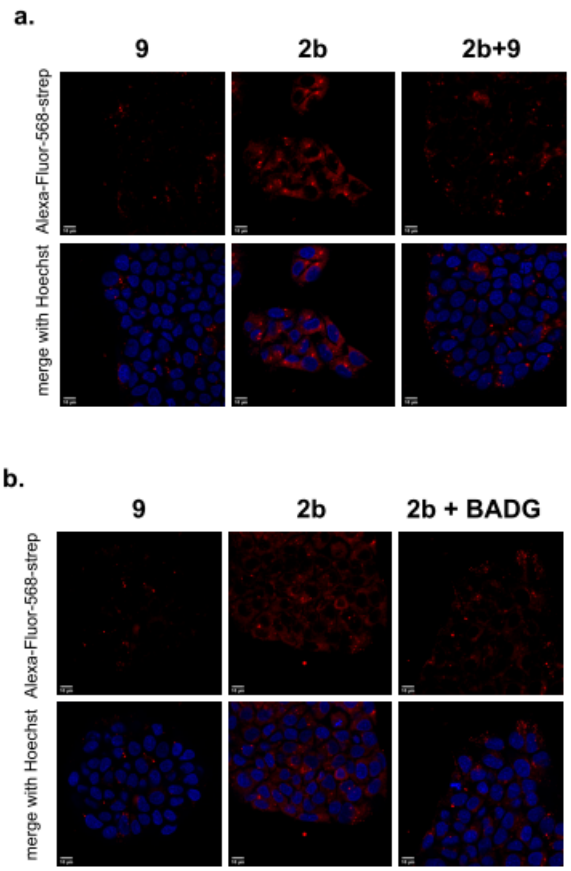

Figure S12: HepG2 cells grown with 500 µM **9** or **2b**, for a inhibition experiment both **9** and **2b** were added simultaneously. **b.** HepG2 cells were grown with 100 µM of **9** or **2b**, Benzyl-2-acetamido-2-deoxy-galactopyranse (BADG) was added with 100 µM after 24h to a culture with **2b** and the cells were analysed after a total culture time of 72h. IEDDA reaction was performed with 6-methyl-tetrazine-peg4-biotin, followed by staining with Alexa-Fluor-568-streptavidin (red) and Hoechst (blue).

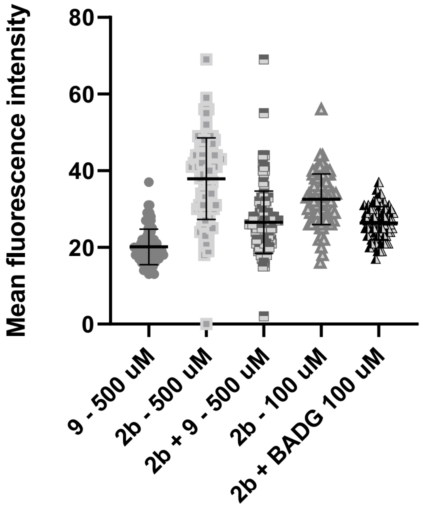

Figure S13: Mean Fluorescence intensity of single cells, after incorporation of control **9** or **2b**, in concentrations of 500 or 100 µM, competition experiment with **9** and **2b**, in the presence of inhibitor Benzyl-2-acetamido-2-deoxy-galactopyranse (BADG). 7-8 pictures from different positions in the well were analyzed.

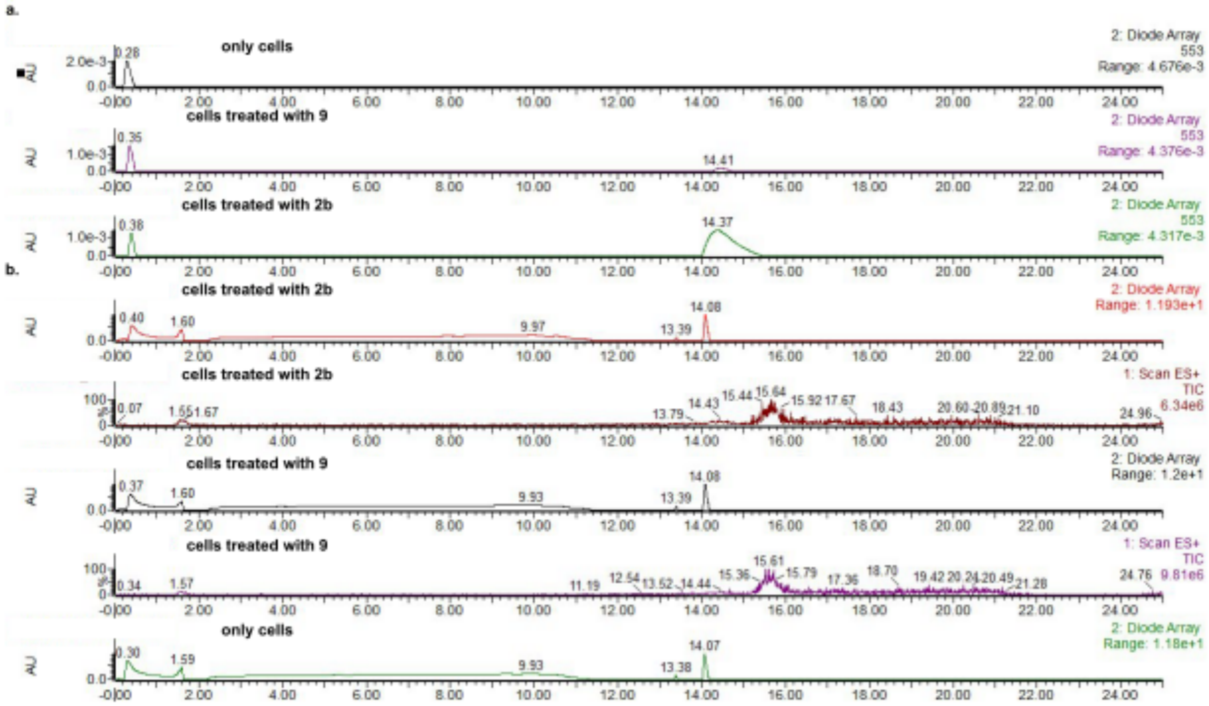

Figure S14: Analysis of cell surface glycans released by tryptic digestion and labelled with 6-methyl-tetrazine-sulfo-Cy3, **a.** Analysis at 553 nm, representing specific tetrazine absorbance **b.** UV (200-650 nm) and ES+ trace of all samples.

2. General Procedures

The used reagents were purchased from Alfa Aesar, Carbosynth Limited, Fisher Scientific and Sigma Aldrich and were used without further purification. Purification of the compounds was performed by chromatography using Silica Gel 60 (mesh 230-400) from Material Harvest. Thin layer chromatography (TLC) was carried out on silica gel coated aluminium plates (60 F_{254}, Merck) and the reactions were visualized with 5% sulfuric acid in ethanol and UV light (λ =254 nm). Proton (^{1}H NMR), carbon (^{13}C NMR) nuclear magnetic resonance spectra were recorded on a Bruker 500 MHz DCM Cryoprobe or 400 MHz DPX-400 Dual spectrometer. All spectra were fully assigned using COESY, HSQC and HMBC, the chemical shifts were quoted on the δ scale in ppm and the solvent peak ($CDCl_3$: ^{1}H = 7.26 ppm, ^{13}C = 77.16 ppm, D_2O: ^{1}H = 4.79 ppm) was used as internal standard. Coupling constants *J* were reported in Hz, using the following splitting abbreviations: s = singlet, d = duplet, t = triplet, dd = duplet from duplet, m = multiplet. High resolution mass spectrometry (HRMS) were received from a Thermo Finnigan Orbitrap Classic using positive ion electronstpray ionization (ESI) for essential compounds.

2.1. Chemical Synthesis

2.1.1. Synthesis of 2-*O*-allyl-1,3,4,6-tetra-*O*-acetly-galactopyranose (2a)

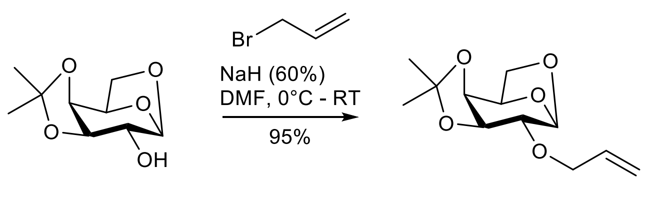

1,6-Anhydro-3,4-isopropylgalactopranose (300 mg, 1.48 mmol) was dissolved in 6 mL anhydrous DMF and cooled to 0 °C. NaH (60% suspension in mineral oil, 149 mg, 4.45 mmol) was added and the reaction was stirred for 30 min. Allylbromid (0.387 mL, 4.45 mmol) was added slowly and the reaction was stirred over night from 0 °C – RT. Water was added carefully. The reaction mixture was extracted with CH_2Cl_2, the organic layer was washed three times with 5 mL of

saturated sodium chloride solution, dried with $MgSO_4$, filtrated through a cotton patch and concentrated. The product 1,6-anhydro-2-allyl-3,4-isopropylgalactose was obtained after column chromatography (petrol/EtOAc 3:1) with a yield of 95% (339.8 mg, 1.4 mmol).

^{1}H-NMR (300 MHz, $CDCl_3$): δ = 5.88 (ddt, J = 17.2 Hz, J =10.4 Hz, J = 5.6 Hz, 1H, C***H***=CH_2), 5.38 (s, 1H, ***H***1), 5.28 (dq, J = 17.2, J = 1.5 Hz, 1H, CH=C***H₂***), 5.19 (ddd, J = 10.4 Hz, J = 2.7 Hz, J = 1.2 Hz, 1H, CH=C***H₂***), 4.46 (t, J = 5.6 Hz, 1H, ***H***3), 4.40 (t, J = 6.4 Hz, 1H, ***H***5), 4.14 (dd, J = 5.9, J = 4.9 Hz, 1H, ***H***4), 4.12 – 4.06 (m, 2H, ***H***6), 4.06 – 4.00 (m, 1H, C***H₂***-CH), 3.53 (dt, J = 10.2, J = 4.2 Hz, 1H, C***H₂***-CH), 3.48 (s, 1H, ***H***2), 1.49 (s, 3H, C***H₃***), 1.32 (s, 3H, C***H₃***) ppm.

^{13}C-NMR (75 MHz, $CDCl_3$): δ = 133.81 (***C***H=CH_2), 117.79 (CH=***C***H_2), 108.36 (***C***(CH_3)$_2$), 99.60 (***C***1), 76.56 (***C***2), 74.05 (***C***4), 71.92 (***C***3), 71.06 (***C***6), 69.21 (***C***5), 62.89 (O***C***H_2-CH=CH_2), 25.66 (***C***H_3), 24.17 (***C***H_3) ppm.

HRMS-ESI+ (m/z): calculated [M + H^+] = 243.1227, found [M + H^+] = 243.1240

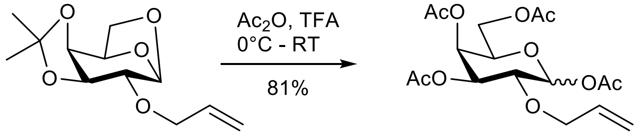

The obtained material (339.8 mg, 1.4 mmol) was dissolved in Ac_2O (7.5 mL/ mmol) and cooled to 0 °C. Trifluoracetic acid (TFA, 1.26 mL/mmol) was added dropwise and the reaction was stirred overnight from 0 °C – RT. The reaction was diluted with EtOAc (10 mL) and washed with saturated solution of sodium bicarbonate (3 x 10 mL) and saturated sodium chloride solution (2 x 10 mL). The organic layer was dried with $MgSO_4$, filtrated through a cotton patch and concentrated. The crude mixture was purified by column chromatography (petrol/ EtOAc 3:1 -> 1:1) and the product 2-allyl-1,3,4,6-*O*-acetyl-galactopyranose was obtained with a yield of 81% (444.3 mg, 1.14 mmol).

^{1}H-NMR (400 MHz, $CDCl_3$): δ = 6.39 (d, J = 3.6 Hz, 1H, ***H***1α), 5.82 (ddd, J = 22.8 Hz, J = 10.8 Hz, J = 5.6 Hz, 1H, C***H***=CH_2), 5.61 (d, J = 8.2 Hz, 1H, ***H***1β), 5.46 (d, J = 2.2 Hz, 1H, ***H***4α), 5.39 (d, J = 2.7 Hz, 1H, ***H***4β), 5.26 (dd, J = 13.5 Hz, J = 2.4 Hz, 1H, CH=C***H₂***), 5.22 (dd, J = 6.6 Hz, J = 3.3 Hz, 1H, ***H***3α), 5.19 (dd, J = 10.5 Hz, J = 1.2 Hz, 1H, CH=C***H₂***), 4.99 (dd,

J = 10.1 Hz, J = 3.4 Hz, 1H, ***H***3β), 4.28 (t, J = 6.7 Hz, 1H, ***H***5), 4.10 – 4.04 (m, 4H, ***H***6a/b, OC***H***$_2$), 3.87 (dd, J = 10.6 Hz, J = 3.6 Hz, 1H, ***H***2α), 3.68 (dd, J = 10.1 Hz, J = 8.2 Hz, 1H, ***H***2β), 2.15, 2.14, 2.03, 2.02 (4 × s, 4 × 3H, C***H***$_3$CO) ppm.

^{13}C-NMR (100 MHz, CDCl$_3$): δ = 170.6, 170.3, 170.2, 169.4 (4 × CH$_3$***C***O), 134.2 (***C***H=CH$_2$), 117.9 (CH=***C***H$_2$), 90.2 (***C***1), 72.3 (***C***2), 72.2 (O***C***H$_2$), 69.5 (***C***3), 68.7 (***C***5), 67.9 (***C***4), 61.5 (***C***6), 21.1, 20.9, 20.8, 20.7 (4 × ***C***H$_3$CO) ppm.

HRMS-ESI+ (m/z): calculated [M + H$^+$] = 389.1442, found [M+ H$^+$] = 389.1440

2.1.2. Synthesis of 2-*O*-allyl-galactopyranose (3a)

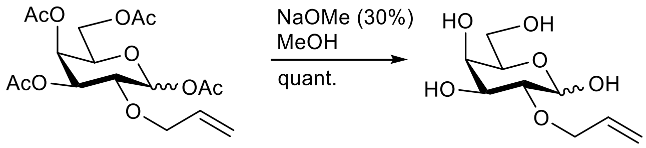

The fully acetylated sugar 2-allyl-1,3,4,6-tetra-*O*-acetyl-galactopyranose (100 mg, 0.257 mmol) was dissolved in 2 mL MeOH and 30% NaOMe solution (0.142 mL) was added. The reaction was stirred at RT until TLC showed complete consumption of the starting material. The mixture was neutralized with Dowex 50W (H$^+$ from), filtrated and concentrated. The final, unprotected monosaccharide was purified by chromatography (EtOAc/MeOH 3:1) and obtained in quantitative yield as mix of both anomers.

^{1}H-NMR (400 MHz, D$_2$O): δ = 6.06 – 5.92 (m, 2H, C***H***=CH$_2$, C***H'***=CH$_2$), 5.44 (d, J = 3.7 Hz, 1H, ***H***1α), 5.41 – 5.25 (m, 4H, CH=C***H***$_2$, CH=C***H'***$_2$) , 4.64 (d, J = 7.9 Hz, 1H, ***H***1β), 4.34 (ddd, J = 39.2 Hz, J = 12.1 Hz, J = 6.2 Hz, 2H, ***H***6'), 4.23 – 4.15 (m, 2H, OC***H***$_2$), 4.07 (dd, J = 11.8 Hz, J = 5.5 Hz, 1H, ***H***3'), 4.00 (d, J = 2.9 Hz, 1H, ***H***3'), 3.97 – 3.88 (m, 1H, ***H***4), 3.79 – 3.71 (m, 2H, ***H***6), 3.71 – 3.64 (m, 2H, ***H***3, ***H***2'), 3.39 (dd, J = 9.9 Hz, J = 8.4 Hz, 1H, ***H***2) ppm.

^{13}C NMR (101 MHz, D$_2$O): δ = 134.1 (***C***H=CH$_2$), 118.5 (CH=***C***H$_2$), 96.4 (***C***1), 90.3 (***C***1'), 79.9 (***C***2), 75.6 (***C***2'), 73.9 (***C***6'), 72.4 (***C***3), 71.5 (O***C***H$_2$), 70.2 (***C***3'), 69.3 (***C***5), 68.8 (***C***4), 61.1(***C***6, ***C***6'), 60.9 (***C***6, ***C***6') ppm.

Signals assigned with ‘ represent the alpha-conformer.

2.1.3. Synthesis of 2-*O*-pentenyl-1,3,4,6-tetra-*O*-acetyl-galactopyranose (2b)

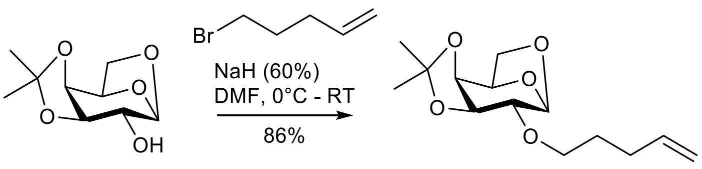

1,6-Anhydro-3,4-isopropylgalactopranose (300 mg, 1.48 mmol) was dissolved in 6 mL anhydrous DMF and cooled to 0 °C. NaH (60% suspension in mineral oil, 149 mg, 4.45 mmol) was added and the reaction was stirred for 30 min. 5-Bromo-1-pentene (0.387 mL, 4.45 mmol) was added slowly and the reaction was stirred over night from 0 °C – RT. Water was added carefully. The reaction mixture was extracted with CH_2Cl_2, the organic layer was washed three times with 5 mL of saturated sodium chloride solution, dried with $MgSO_4$, filtrated through a cotton patch and concentrated. The product 1,6-anhydro-2-pentenyl-3,4-isopropylgalactose was obtained after column chromatography (petrol/EtOAc 3:1) with a yield of 86% (343.8 mg, 1.27 mmol).

^{1}H-NMR (500 MHz, $CDCl_3$): δ = 5.80 (ddt, $J_{CH/CH2=CH}$ = 16.9 Hz, $J_{CH/CH2=CH}$ = 10.2 Hz, $J_{CH/CH2}$ = 6.7 Hz, 1H, C***H***=CH$_2$), 5.41 (d, $J_{H1/H2}$ = 1.3 Hz, 1H, ***H***1), 5.03 (dq, $J_{CH2=CH/CH}$ = 17.1 Hz, $J_{CH2=CH/CH2=CH}$ = 1.7 Hz, 1H, CH=C***H***$_2$), 4.97 (m,1H, CH=C***H***$_2$), 4.50 (t, $J_{H6/H6,5}$ = 5.7 Hz, 1H, ***H***6), 4.46 – 4.41 (m, 1H, ***H***4), 4.16 (dt, $J_{H3/H4}$ = 7.2 Hz, $J_{H3/H1}$ = 1.1 Hz, 1H, ***H***3), 4.08 (d, $J_{H6/H5}$ = 7.5 Hz, 1H, ***H***6), 3.64 – 3.60 (m, 1H, ***H***5), 3.60 – 3.55 (m, 2H, O-C***H***$_2$), 3.43 (d, $J_{HH2/H1}$ = 0.9 Hz, 1H, ***H***2), 2.13 (m, 2H, C***H***$_2$-CH=CH$_2$), 1.70 (m, 2H, C***H***$_2$-CH$_2$-O), 1.53 (s, 3H, C***H***$_3$), 1.36 (s, 3H, C***H***$_3$) ppm.

^{13}C NMR (120 MHz, $CDCl_3$): δ = 138.1 (***C***H=CH$_2$), 115.2 (CH=***C***H$_2$), 99.9 (***C***1), 78.3 (***C***2), 74.3 (***C***3), 72.2 (***C***6), 69.5 (***C***6), 63.2 (***C***H$_2$O), 31.1, 30.3 (***C***H$_2$-CH=CH$_2$), 28.9 (***C***H$_2$-CH$_2$O), 25.9 (***C***H$_3$), 24.5 (***C***H$_3$) ppm.

HRMS-ESI+ (m/z): calculated [M + H$^+$] = 271.1540, found [M + H$^+$] = 271.1546

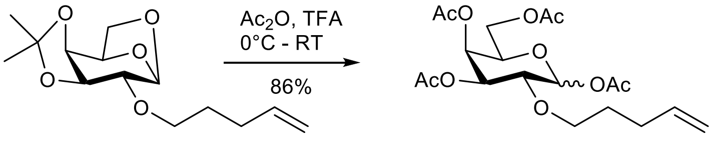

The obtained material (343.8 mg, 1.27 mmol) was dissolved in Ac_2O (7.5 mL/ mmol) and cooled to 0 °C. Trifluoracetic acid (TFA, 1.26 mL/mmol) was added dropwise and the reaction was stirred overnight from 0 °C – RT. The reaction was diluted with EtOAc (10 mL) and washed with saturated solution of sodium bicarbonate (3 x 10 mL) and saturated sodium chloride solution (2 x 10 mL). The organic layer was dried with $MgSO_4$, filtrated through a cotton patch and concentrated. The crude mixture was purified by column chromatography (petrol/ EtOAc 3:1 -> 1:1) and the product 2-pentenyl-1,3,4,6-*O*-acetyl-galactopyranose was obtained with a yield of 86% (455.6 mg, 1.09 mmol).

^{1}H-NMR (400 MHz, $CDCl_3$): δ = 6.41 (d, *J* = 3.6 Hz, 1H, ***H***1α), 5.77 (ddt, *J* = 16.9 Hz, *J* = 10.1 Hz, *J* = 6.7 Hz, 1H, C***H***=CH_2), 5.59 (d, *J* = 8.1 Hz, 1H, ***H***1β), 5.46 (d, *J* = 2.4 Hz, 1H, ***H***4α), 5.38 (d, *J* = 3.1 Hz, 1H, ***H***4β), 5.20 (dd, *J* = 10.5 Hz, *J* = 3.2 Hz, 1H, ***H***3α), 5.04 – 4.92 (m, 3H, ***H***3β, CH=C***H***$_2$), 4.28 (t, *J* = 6.6 Hz, 1H, ***H***5α), 4.07 (dd, *J* = 6.7 Hz, *J* = 2.0 Hz, 2H, ***H***6a/b), 3.78 (dd, *J* = 10.5 Hz, *J* = 3.6 Hz, 1H, ***H***2α), 3.63 (ddd, *J* = 15.2 Hz, *J* = 11.1 Hz, *J* = 4.6 Hz, 2H, ***H***2β, OC***H***$_2$), 3.48 (dt, *J* = 9.1 Hz, *J* = 6.5 Hz, 1H, OC***H***$_2$), 2.15 (2 × s, 6H, C***H***$_3$), 2.06 (d, *J* = 5.3 Hz, 2H, OCH_2CH_2C***H***$_2$), 2.03, 2.02 (2 × s, 6H, C***H***$_3$), 1.63 – 1.55 (m, 2H, OCH_2C***H***$_2$) ppm.

^{13}C-NMR (100 MHz, $CDCl_3$): δ = 170.7, 170.4, 170.3, 169.4 (4 × CH_3***C***O), 138.1 (***C***H=CH_2), 115.1 (CH=***C***H_2), 94.1 (***C***1β), 90.1 (***C***1) 73.2 (***C***2), 70.9 (O***C***H_2), 69.4 (***C***3), 68.7 (***C***5), 67.9 (***C***3), 61.5 (***C***6), 29.9 (OCH_2CH_2***C***H_2), 29.1 (OCH_2***C***H_2), 21.1, 20.9, 20.8, 20.7 (4 × ***C***H_3CO) ppm.

HRMS-ESI+ (m/z): calculated [M + Na^+] = 439.1575, found [M + Na^+] = 439.1571

2.1.4. Synthesis of 2-*O*-pentenyl-galactopyranose (3b)

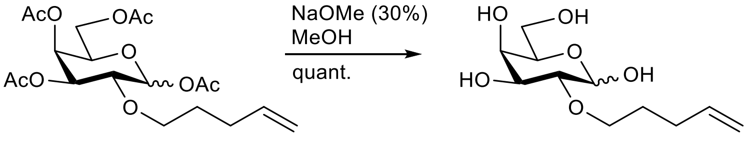

2-*O*-pentenyl-1,3,4,6-*O*-acetyl-galactopyranose (100 mg, 0.24 mmol) was dissovled in 2 mL MeOH and 30% NaOMe solution (0.136 mL) was added. The reaction was stirred at RT until TLC showed complete consumption of the starting material. The mixture was neutralized with Dowex 50W (H^+ form), filtrated and concentrated. The final, unprotected monosaccharide was purified by chromatography (EtOAc/MeOH 3:1) and was obtained in quantitative yield.

^{1}H NMR (400 MHz, D_2O) δ = 6.00 – 5.85 (m, 2H, C***H***=CH_2, C***H’***=CH_2), 5.44 (d, *J* = 3.5 Hz, 1H, ***H***1α), 5.10 (d, *J* = 17.3 Hz, 2H, CH=C$\boldsymbol{H}_2$, CH=C$\boldsymbol{H'}_2$), 5.03 (d, *J* = 10.2 Hz, 2H, CH=C$\boldsymbol{H}_2$, CH=C$\boldsymbol{H'}_2$), 4.62 (d, *J* = 7.9 Hz, 1H, ***H***1β), 4.08 (dd, *J* = 11.5 Hz, *J* = 5.3 Hz, 1H, ***H***5’), 3.96 (dd, *J* = 25.6 Hz, *J* = 2.7 Hz, 2H, ***H***4, ***H***5), 3.92 – 3.84 (m, 2H, ***H***3α, OC$\boldsymbol{H}_2$), 3.82 – 3.63 (m, 10H, OC$\boldsymbol{H}_2$, OC$\boldsymbol{H'}_2$, ***H***3’, ***H***4’, ***H***6a,b, ***H***6’a,b), 3.60 (dd, *J* = 10.3 Hz, *J* = 3.9 Hz, 1H, ***H***2’), 3.35 – 3.27 (m, 1H, ***H***2), 2.15 (q, *J* = 7.0 Hz, 4H, C$\boldsymbol{H}_2$CH=CH_2, C$\boldsymbol{H'}_2$CH=CH_2), 1.76 – 1.67 (m, 4H, OCH_2C$\boldsymbol{H}_2$, OCH_2C$\boldsymbol{H'}_2$) ppm.

^{13}C NMR (101 MHz, D_2O) δ = 139.0, 138.9 (***C***H=CH_2, ***C***’H=CH_2), 114.7 (CH=$\boldsymbol{C}H_2$), 96.3 (***C***1β), 90.2 (***C***1α), 80.3 (***C***2), 76.3 (***C***2’), 75.0 (***C***3), 72.6 (O$\boldsymbol{C}H_2$), 72.4 (***C***3/***C***4’), 70.2 (***C***5’), 70.0 (O$\boldsymbol{C}H_2$), 69.3 (***C***5), 68.8 (***C***4), 68.4 (***C***3’), 61.14, 60.89 (***C***6, ***C***6’) 29.4 ($\boldsymbol{C}H_2$CH=CH_2), 28.2 ($OCH_2$$\boldsymbol{C}H_2$) ppm.

2.1.5. Synthesis of 6-*O*-pentenyl-1,2,3,4-tetra-*O*-acetyl-galactopyranose (5)

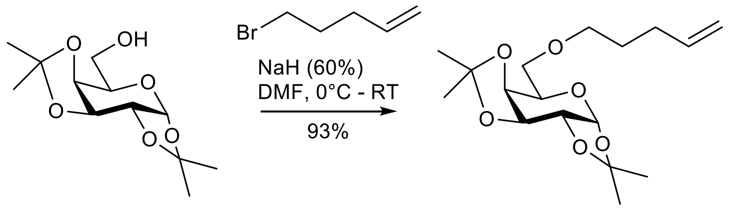

The monosaccharide 1,2,3,4-diisopropyl-galactopyranose (300 mg, 1.15 mmol) was dissovled in 5 mL anhydrous DMF and cooled to 0 °C. NaH (60% suspension in mineral oil, 116 mg, 3.46 mmol) was added and the mixture was stirred for 30 min. 5-Bromo-1-pentente (0.409 mL, 3.46 mmol) was added slowly to the reaction and the mixture was stirred overnight from 0 °C to

RT. 2 mL of water were added carefully to the reaction and the mixture was extracted with CH_2Cl_2 (3 x 5 mL). The organic layer was washed with saturated sodium chloride solution (3 x 5 mL), dried with $MgSO_4$, filtrated through a cotton patch and concentrated. The crude mixture was purified by chromatography (petrol/EtOAc 3:1 -> 1:1) and the product 6-*O*-pentenyl-1,2,3,4—diisopropyl-galactopyranose was obtained with a yield of 93% (350 mg, 1.07 mmol)

^{1}H-NMR (400 MHz, $CDCl_3$): δ = 5.87 – 5.74 (m, 1H, C***H***=CH_2), 5.53 (d, J = 5.0 Hz, 1H, ***H***1), 5.05-4.97 (m, 1H, CH=C***H***$_2$), 4.97-4.91 (m, 1H, CH=C***H***$_2$), 4.59 (dd, J = 7.9 Hz, J = 2.3 Hz, 1H, ***H***3), 4.29 (dd, J = 5.0 Hz, J = 2.4 Hz, 1H, ***H***2), 4.25 (dd, J = 7.9 Hz, J = 1.8 Hz, 1H, ***H***4), 3.95 (td, J = 6.3 Hz, J = 1.8 Hz, 1H, ***H***5), 3.66 – 3.53 (m, 2H, ***H***6), 3.53 – 3.44 (m, 2H, OC***H***$_2$$CH_2$), 2.15-2.06 (m, 2H, C***H***$_2$CH=CH_2), 1.67 (dt, J = 13.7 Hz, J = 6.7 Hz, 2H, OCH_2C***H***$_2$), 1.53, 1.44,1.33, 1.32 (4 × s, 4 × 3H C***H***$_3$) ppm.

^{13}C-NMR (100 MHz, $CDCl_3$): δ = 138.5 (***C***H=CH_2), 114.8 (CH=***C***H$_2$), 109.3, 108.6 (2 × ***C***$(CH_3)_2$), 96.5 (***C***1), 71.3 (***C***4), 70.9, 70.8 (***C***2, ***C***3, O***C***H$_2$$CH_2$), 69.4 (***C***6), 66.8 (***C***5), 30.4 (***C***H$_2$CH=CH_2), 28.9 (OCH_2***C***H$_2$), 26.2, 26.1, 25.1, 24.6 (4 × ***C***H$_3$) ppm.

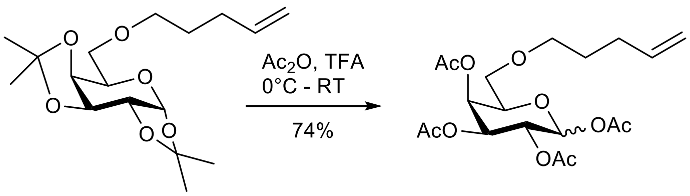

The sugar 6-*O*-pentenyl-1,2,3,4-diisopropyl-galactopyranose (350 mg, 1.07 mmol) was dissolved in Ac_2O (8.33 mL, 7.5 mL/mmol) and cooled to 0 °C. TFA (1.39 mL, 1.26 mL/mmol) was added dropwise and the reaction was stirred from 0 °C-RT overnight. The reaction was diluted with EtOAc and the organic layer was washed with saturated solution of sodium bicarbonate (3 x 5 mL), saturated sodium chloride solution (2 x 5 mL), dried with $MgSO_4$, filtrated and concentrated. The crude mixture was purified by chromatography (petrol/EtOAc 4:1 -> 2:1) and the product was obtained with a yield of 74% (0.79 mmol, 329 mg).

^{1}H-NMR (500 MHz, $CDCl_3$): δ = 6.35 (d, J = 2.7 Hz, 1H, ***H***1), 6.08 (d, J = 1.5 Hz, 1H, ***H***1'), 5.82-5.70 (m, 1H, C***H***=CH_2), 5.53 (s, 1H, ***H***3), 5.32 (t, J = 2.8 Hz, 1H, ***H***2), 4.99 (dd, J = 17.1 Hz,

$J = 1.4$ Hz, 1H, CH=C***H***$_2$), 4.93 (d, $J = 10.2$ Hz, 1H, CH=C***H***$_2$), 4.24 (dd, $J = 13.3$ Hz, $J = 6.8$ Hz, 1H, ***H***4), 3.49 – 3.29 (m, 5H, ***H***5, ***H***6a/b, OC***H***$_2$CH$_2$), 2.13 (s, 6H, 2 × C***H***$_3$CO), 2.07 (dd, $J = 5.2$ Hz, $J = 2.6$ Hz, 2H, C***H***$_2$CH=CH$_2$), 2.00, 1.98 (2 × s, 6H, 2 × C***H***$_3$CO), 1.63 – 1.56 (m, 2H CH$_2$C***H***$_2$CH$_2$) ppm.

^{13}C-NMR (126 MHz, CDCl$_3$): δ = 170.3, 170.2, 170.1, 169.2 (4 × CH$_3$***C***O), 138.2 (***C***H=CH$_2$), 114.9 (CH=***C***H$_2$), 89.9 (***C***1), 71.2 (O***C***H$_2$CH$_2$), 70.1 (***C***4), 68.4 (***C***6), 68.1 (***C***3), 67.7, 66.8 (***C***2), 30.1 (***C***H$_2$CH=CH$_2$), 28.7 (CH$_2$***C***H$_2$CH$_2$), 20.8, 20.7, 20.6, 20.5 (4 × ***C***H$_3$CO) ppm.

HRMS-ESI+ (m/z): calculated [M + Na$^+$] = 439.1575, found [M + Na$^+$] = 439.1571

2.1.6. Synthesis of 6-*O*-pentenyl-galactopyranose (6)

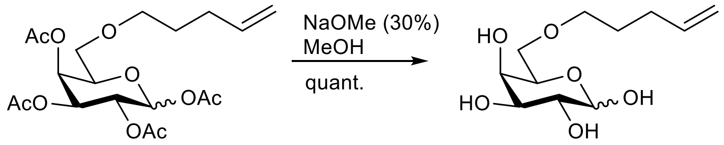

6-*O*-pentenyl-1,2,3,4-tetra-*O*-acetyl-galactopyranose (100 mg, 0.24 mmol) was dissovled in 2 mL MeOH and 30% NaOMe solution (0.136 mL) was added. The reaction was stirred at RT until TLC showed complete consumption of the starting material. The mixture was neutralized with Dowex 50W (H$^+$ form), filtrated and concentrated. The final, deprotected monosaccharide 6-*O*-pentenyl-galactopyranose was obtained after chromatography (EtOAc/MeOH 3:1) in quantitative yield.

^{1}H-NMR (400 MHz, D$_2$O): δ = 5.92 (ddt, $J = 16.9$ Hz, $J = 10.4$ Hz, $J = 6.6$ Hz, 2H, C***H***=CH$_2$, C***H’***=CH$_2$), 5.26 (d, $J = 3.5$ Hz, 1H, ***H***1α), 5.09 (t, $J = 12.3$ Hz, 2H, CH=C***H***$_2$, CH=C***H’***$_2$), 5.03 (d, $J = 10.2$ Hz, 2H, CH=C***H***$_2$, CH=C***H’***$_2$), 4.58 (d, $J = 7.8$ Hz, 1H, ***H***1β), 4.24 – 4.18 (m, 1H, ***H***5’), 3.97 (s, 1H, ***H***5), 3.92 (d, $J = 3.1$ Hz, 1H, ***H***4), 3.86 (dd, $J = 10.6$ Hz, $J = 3.1$ Hz, 1H, ***H***3’), 3.81 (dd, $J = 9.5$ Hz, $J = 4.3$ Hz, 1H, ***H***2’), 3.69 (t, $J = 5.5$ Hz, 2H, ***H***6a,b), 3.67 – 3.62 (m, 1H, ***H***3), 3.62 – 3.54 (m, 2H, OC***H***$_2$), 3.53 – 3.46 (m, 1H, ***H***2), 2.13 (dd, $J = 13.9$ Hz, $J = 6.9$ Hz, 4H, C***H***$_2$CH=CH$_2$, C***H’***$_2$CH=CH$_2$), 1.76 – 1.65 (m, 4H, OCH$_2$C***H***$_2$, OCH$_2$C***H’***$_2$) ppm.

^{13}C-NMR (101 MHz, D_2O): δ = 138.8 (***C***H=CH_2), 114.7 (CH=***C***H_2), 96.4 (***C***1β), 92.3 (***C***1α), 73.3 (***C***2'), 72.7 (***C***3), 71.8 (***C***2), 70.8 (O***C***H_2), 69.7 (***C***6), 69.6 (***C***5), 69.1 (***C***3'), 69.0 (***C***4), 68.6 (***C***5), 29.5 (***C***H_2CH=CH_2), 27.7 (OCH_2***C***H_2) ppm.

2.2. Kinetic Studies

The kinetic studies were performed in aqueous PBS buffer at pH = 7.4 as solvent. The reaction progress was monitored by following the decrease in tetrazine absorption at 530 nm. The optimal tetrazine concentration was determined by an absorbance screen, determining a concentration of 0.6 mM 6-methyl-tetrzaine-amine as optimal concentration per well. A stock solution of 20 mM was prepared of every de-acetylated galactose derivative, from which further dilutions of 16 mM, 12 mM, 8 mM and 4 mM were prepared. The solutions of galactose derivatives and 6-methyl-tetrazine-amine were mixed to a final volume of 100 µL in 96-well plates and the decline in absorption at 530 nm was followed for 16 h in a microplate reader at 37 °C. The pseudo-first order rate constant k_{obs} was calculated for each concentration using GraphPad Prism 6.01 with an exponential decay function. The corresponding second order rate constant k_2 for each galactose derivative was calculated based on the concentration dependent values for k_{obs} and the resulting linear function.

2.3. Cell Culture

All cell lines were maintained in a humidified incubator at 37°C under 5% CO_2 and split before reaching confluence using TrypLE™ Express. All cell lines were grown in DMEM medium (high glucose) supplemented with 10% heat-inactivated FBS, 2 mM GlutaMAX™, 10 mM HEPES, 1% NEAA, 100 units/mL penicillin and 100 µg/mL streptomycine, further named complete medium (cDMEM). All reagents were bought from Gibco, Life Technologies (USA).

2.3.1. Cell toxicity

The toxicity of the galactose derivatives was assessed using a CellTiter-Blue^R Cell Viability Assay (Promega, USA). In this approach the conversion of the dye reaszurine into the fluorescent resorufin product by metabolically active cells is measured. Cells were seeded at a concentration of 10000 cells/ well (100 µl) for 48 h or 5000 cells/well (100 µl) for 72 h timepoints in a flat-bottom 96 well-plate and allowed to adhere and adapt to the plates for 24 h. After this, the cell culture medium was exchanged to complete medium supplemented with 100 µM or 200 µM of the corresponding galactose derivative. Each concentration was assed in technical triplicates. Plates were incubated for 48 h or 72 h after which the cell viability was determined by exchanging the culture medium to medium supplemented with CellTiter-Blue Reagent (dilution 1:20 from commercial stock). The plates were incubated for additional 1 h 30 min with the reagent, before analyzing the fluorescence intensity on a Infinite M200 (Tecan, USA) plate reader (λ_{exc} = 530, λ_{em} = 590). Relative fluorescence units were normalized to the values obtained for the appropriate vehicle controls and the results were shown as a percentage of the values obtained from cells cultured with the vehicle.

2.3.2. Metabolic Labeling in Huh7 and HepG2 cells

Cells were seeded with a density of 15000 cells/well (300 µL) on glass coverslips in 24-well plates and allowed to adhere for 24 h. Following this, the cell culture medium was exchanged to complete medium supplemented with 100 µM of the corresponding galactose derivative and the cells were cultured for 72 h. After this, the medium was removed and complete medium containing 25 µg/mL streptavidine (from 1 mg/mL stock in water) was added for 40 min to block endogenous biotin. The solution was aspirated and the cells were washed with PBS (3x, 200 µL/well). The labeling reaction was performed adding complete medium containing 200 µM 6-methyl-tetrazine-peg4-biotin (Jena Bioscience, stock of 1.5 mM in DMSO) to the cells and allow the reaction to occure for 5 h at 37 °C in the incubator. The tetrazine containing media was removed and the cells were washed with PBS (3x, 200 µL/well), followed by adding 6.6 µg/mL Alexa-Fluor-568-streptavidine (in PBS + 5% FBS, 200 µL/well, 20 min, RT) and Hoechst 33342

(in PBS + 5% FBS, 1:1000, 5 min, RT). For labeling of the cell membrane, CellMask ™ Deep Red Plasma membrane stain (ThermoFisher scientific, in PBS + 5% FBS, 1:1000, 2 min, 37 °C) was applied before proceeding with the fixation protocol.

The cells were fixed using 4% PFA solution for 10 min at RT and the coverslips were mounted on glass objective slides using Fluoromount G™. The cells were analyzed using a LSM880 confocal point-scanning microscope (Zeiss, Germany), equipped with a Diode 405-30 nm (Hoechst) and a DPSS 561-20 nm (Alexa-Fluor-568) laser unit. The pictures were acquired with a 63x Plan-Apochromat Oil objective and processed with ImageJ 1.49v software to remove background noise. Representative images were chosen from 5 different experiments. Quantification of the fluorescence intensity resulting from the incorporated galactose derivatives and labeling with 6-methyl-tetrazine-peg4-biotin and Alexa-Fluor-568-streptavidine was done using ImageJ 1.49v software by selecting the individual cells per picture as region of interest and comparing the intensity. The values were presented as ratio to the intensity of the corresponding negative control.

The labeling experiments were performed in the same way using acetyl protected galactose derivatives or deprotected derivatives.

2.3.3. Metabolic labeling of HepG2 cells in presence of inhibitors for GLUT1

HepG2 cells were seeded in 24-well plates, either on glass coverslips (15000/well) for microscopy analysis or on the plain plate (50000/well) for analysis by flow cytometry. The cells were allowed to adhere for 24h, before the medium was changed to complete medium containing 100 µM of the unnatural galactose derivative and different concentrations of the inhibitor (10 µM of WZB117, STF31 and cytochalasine B for microscopy studies; 10 µM, 20 µM and 30 µM of WZB117 for flow cytometry). The stock solutions of all inhibitors were prepared in DMSO at a concentration of 20 mM for WZB117 and STF31, as well as 2 mM for Cytochalasine B. The cells were grown for 72 h in the presence of the unnatural galactose derivative and the inhibitors, before being analyzed by confocal point scanning microscopy or flow cytometry.

For microscopy analysis, the medium was removed and complete medium containing 25 µg/mL streptavidine (from 1 mg/mL stock in water) was added for 40 min to block endogenous biotin.

The solution was aspirated and the cells were washed with PBS (3x, 200 µL/well). The labeling reaction was performed adding complete medium containing 200 µM 6-methyl-tetrazine-peg4-biotin (Jena Bioscience, stock of 1.5 mM in DMSO) to the cells and allow the reaction to occure for 5 h at 37 °C in the incubator. The tetrazine containing media was removed and the cells were washed with PBS (3x, 200 µL/well), followed by adding 6.6 µg/mL Alexa-Fluor-568-streptavidine (in PBS + 5% FBS, 200 µL/well, 20 min, RT) and Hoechst 33342 (in PBS + 5% FBS, 1:1000, 5 min, RT). The cells were fixed using 4% PFA solution for 10 min at RT and the coverslips were mounted on glass objective slides using Fluoromount G^{TM}. The cells were analyzed using a LSM880 confocal point-scanning microscope (Zeiss, Germany), equipped with a Diode 405-30 nm (Hoechst) and a DPSS 561-20 nm (Alexa-Fluor-568) laser unit. The pictures were acquired with a 63x Plan-Apochromat Oil objective and processed with ImageJ 1.49v software to remove background noise and quantify the mean fluorescence intensity per cell of the sugar derived labeling.

For analysis by flow cytometry, the medium was aspirated after 72 h and complete medium containing 25 µg/mL streptavidin (from 1 mg/mL stock in water) was added for 40 min at 37 °C to block endogenous biotin. The cells were rinsed with PBS (3 x 200 µL/well) and complete medium containing 200 µM 6-methyl-tetrazine-peg4-biotin was added for 5 h at 37 °C. The medium was removed and the cells were detached with 20 mM EDTA solution (5 min, 37°C), collected by centrifugation (2000 g, 5 min) and washed with PBS + 5% FBS (100 µl per cell pellet). 6.6 µg/mL Alexa-Fluor-568-streptavidin in PBS + 5% FBS (100 µL per cell pellet) was added for 20 min at RT, followed by washing the cell pellet with PBS + 5% FBS (3x100 µL per cell pellet). The cells were fixed with 4% PFA solution (8 min, RT) and resuspended in 300 µL PBS + 5% FBS for analysis. The cells were analyzed in a BD LSRFortessa X-20 cell analyzer, using a 561 nm laser (Alexa-Fluor-568). The results were analyzed using FACSDiva, FLOWJOW and GraphPad Prism software packages.

2.3.4. Metabolic labeling of HepG2 cells for cell lysis and pull-down

HepG2 cells were seeded in T75 flasks (1.5*106 cells/flask) in DMEM medium (high glucose), supplemented with 10% heat-inactivated FBS, 1% GlutaMAX, 1% HEPES, 1% NEAA and 100

units/mL penicillin and streptomycine, further named complete medium (cDMEM). The cells were allowed to attache at least 12 h, before the medium was changed to cDMEM supplemented with 100 µM penta-acetyl-galactose (Ac5Gal), 1,3,4,6-tetraacetyl-2-O pentenyl-galactose (2OPent) or 1,2,3,4-tetraacetyl-6 O pentenyl-galactose (6OPent). The cells were grown for 72 h, before the media was removed and the cells were gently flushed with PBS (1x). The cells were harvested using 1 mL of lysis buffer (20 mM Tris pH = 7.6, 300 mM NaCl, 1% TritonX100, 5% glycerol, 1 mM EDTA, 1:10 protease inhibitor) and incubated on ice for 30 min. The cell lysate was obtained after centrifugation at 14000 g for 10 min at 4°C. 200 µL of whole cell lysate were treated with 50 µM of 6 methyl tetrazine peg4 bioting and the labelling reaction was performed at room temperature overnight. To each sample, 100 µl of a suspension of streptavidin magnetic beads (Pierce Streptavidin Magnetic Beads, 10 mg/mL) was added and the binding was performed at room temperature for 1 h. The supernatant was collected and the magnetic beads were washed twice with PBS (1x). The magnetic beads were suspended in 50 µL 4xSDS loading buffer with 100 µM DTT and boiled for 10 min at 90 °C. The samples were resolved in a 12% SDS gel.

2.3.5. Competition experiment and Inhibition of *O*-glycosylation

HepG2 cells were seeded on glass coverslips in 24-well plates (15000 cells/well) and were allowed to attach. On the next day, the media was changed to cDMEM supplemented with an indicated concentration of **2b**, **5** or **9**. For competition experiments the same concentration of **9** was added to samples with **2b**. For an inhibition of *O*-glycosylation, Benzyl 2-acetamido-2-deoxy-α-d-galactopyranoside (BADG) was added with 100 µM after 24 h of cell growth until the end of a total culturing time of 72 h. After a culturing time of 72 h, the media was removed and endogenous biotin was blocked with 25 µg/mL streptavidin (in 200 µL cDMEM, 30 min @ 37 °C). The samples were washed repeatedly with PBS, before a solution of 200 µM 6-methyl--tetrazine-peg4-biotin in 200 µL cDMEM was added (5 h @ 37 °C). The media was removed and the cells were rinsed with PBS. The staining was performed with 6.6 µg/mL Alexa-Fluor-568-streptavidin in PBS + 3% FBS (30 min @ room temperature) and Hoechst 33342 (1:1000, 8 min @ room temperature). The cells were fixed with 4% paraformaldehyde solution (8 min @ room temperature) and mounted on glass objectives with Fluoromount G™. The pictures were acquired on a LSM880 confocal point-scanning microscope (Zeiss, Germany), equipped with a Diode 405-

30 nm (Hoechst) and a DPSS 562-20 nm (Alexa-Fluor-568) laser unit. A 63x Plan-Apochromat Oil objective was used for acquisition and the pictures were analysed by using ImageJ 1.49v software package. Pictures were acquired from different regions on the coverslips and the fluorescence intensity resulting from incorporated galactose derivatives was quantified by assigning ROIs to single cells.

2.3.6. Release of cell surface glycans

Huh7 cells were seeded in 6-well plates (1x106 cells/ well) in cDMEM and were allowed to attach overnight. On the next day, the media was changed to cDMEM supplemented with 100 µM of **9** or **2b** and the cells were cultured for 72 h. To analyse possible cell surface glycans, the cells were harvested in cDMEM and washed with 500 µL PBS (3x, 600 rpm, 10 min @ 4 °C). The cells were suspended in 500 µL PBS and 10 µL of Trypsin solution (20 µg trypsin in 100 µL acetic acid 50 mM) was added. The reaction was incubated at 37 °C for 15 min, before the supernatant was separated (15000 rpm, 15 min @ 4 °C). The supernatant was collected and trypsin was heat-inactivated @ 98°C for 5 min.

A 5 µL sample of the supernatant was diluted with 5 µL MilliQ water and the reaction was performed with 1 µL 6-Methyl-tetrazine-sulfo-Cy3 solution (1.1 mM in PBS) overnight @ 37 °C. A sample of 1 µL was diluted with 9 µL MilliQ water for analysis in an Acquity LC/MS system.

It was possible to detect a specific peak at 553nm in cells treated with compound **2b** for 72 h.

2.4. Infection Studies

Sporozoites from *Plasmodium berghei*, expressing green fluorescent protein (GFP), were dissected in non-supplemented DMEM medium from the salivary glands of infected female *A. stephensi* mosquitoes, bred at Instituto de Medicina Molecular. In general, HepG2 cells were seeded one day prior to infection to adhere in the cell culture plates.

2.4.1. Infection of HepG2 cells and analysis by confocal microscopy

HepG2 cells were seeded on glass coverslips (15000/well, 300 µL) in 24-well plates and allowed to adhere for 24h in complete medium. On the day of infection, the medium was aspirated and exchanged to complete medium supplemented with 100 µM of the corresponding galactose derivative (from 100 mM stock in DMSO) or the control sugar and Fungizone (1:200, from commercial stock). GFP-expressing sporozoites from *Plasmodium berghei* were dissected in DMEM from salivary glands of infected female *A. stephensi* mosquitoes and 60000 sporozoites per well were added directly to the cells. The plate was centrifuged for 4 min at 200g to ensure simultaneous settling of the sporozoites on the cells. After 2 hpi, the medium was removed carefully and the cells were rinsed with PBS (3x 200 µL/well) to remove mosquito host debris, before adding again complete medium containing 100 µm of the galactose derivative or the control sugar and fungizone. The cells were grown in a humidified incubator at 37 °C with 5% CO_2 until 48 hpi. At this point, the same staining procedure as described in the section above was applied to the cells and the fluorescence intensity resulting from the incorporation of the unnatural galactose derivatives was analyzed in a LSM880 confocal point-scanning microscope (Zeiss, Germany), equipped with a Diode 405-30 nm (Hoechst) and a DPSS 561-20 nm (Alexa-Fluor-568) laser unit. The pictures were acquired with a 63x Plan-Apochromat Oil objective and processed with ImageJ 1.49v software to remove background noise. Representative images were chosen from 5 different experiments. Quantification of the fluorescence intensity resulting from the incorporated galactose derivatives and labeling with 6-methyl-tetrazine-peg4-biotin and Alexa-Fluor-568-streptavidine was done using ImageJ 1.49v software by selecting the individual cells per picture as region of interest and comparing the intensity. The values were presented as ratio to the intensity of the corresponding negative control.

2.4.2. Infection of HepG2 cells and analysis by flow cytometry and imaging flow cytometry

HepG2 cells were seeded (50000/well, 300 µL) in 24-well plates and allowed to adhere for 24h in complete medium. On the day of infection, the medium was aspirated and exchanged to complete

medium supplemented with 100 µM of the corresponding galactose derivative (from 100 mM stock in DMSO) or the control sugar and Fungizone (1:200, from commercial stock). GFP-expressing sporozoites from *Plasmodium berghei* were dissected in DMEM from salivary glands of infected female *A. stephensi* mosquitoes and 60000 sporozoites per well were added directly to the cells. The plate was centrifuged for 4 min at 200g to ensure simultaneous settling of the sporozoites on the cells. After 2 hpi, the medium was removed carefully and the cells were rinsed with PBS (3x 200 µL/well) to remove mosquito host debris, before adding again complete medium containing 100 µm of the galactose derivative or the control sugar and fungizone. The cells were grown in a humidified incubator at 37 °C with 5% CO_2 until 48 hpi. The medium was aspirated and complete medium containing 25 µg/mL streptavidine (from 1 mg/mL stock in water) was added for 40 min at 37 °C to block endogenous biotin. The cells were rinsed with PBS (3 x 200 µL/well) and complete medium containing 200 µM 6-methyl-tetrazine-peg4-biotin was added for 5 h at 37 °C. The medium was removed and the cells were detached with 20 mM EDTA solution (5 min, 37°C), collected by centrifugation (2000 g, 5 min) and washed with PBS + 5% FBS (100 µl per cell pellet). 6.6 µg/mL Alexa-Fluor-568-streptavidin in PBS + 5% FBS (100 µL per cell pellet) was added for 20 min at RT, followed by washing the cell pellet with PBS + 5% FBS (3x100 µL per cell pellet). The cells were fixed with 4% PFA solution (8 min, RT) and resuspended in 300 µL PBS + 5% FBS for analysis. The cells were analyzed in a BD LSRFortessa X-20 cell analyzer, using a 488 nm laser (GFP) and 561 nm laser (Alexa-Fluor-568). The results were analyzed using FACSDiva, FLOWJOW and GraphPad Prism software packages. The data shown result from a pool of at least 3 different experiments. The mean or median fluorescence intensities resulting from the incorporated unnatural galactose derivative (Alexa-Fluor-568) were presented as ratio to the corresponding negative control.

For analysis using Amnis ImageStreamX imaging flow cytometer, the cells were grown, infected and stained in the same way as described above. The final volume of the cells in PBS + 5% FBS was decreased to 100 µL. A 488nm laser (GFP) and 561 nm laser (Alexa-Fluor-568) were used for the analysis, as well as bright field light. A typical acquisition setting is represented below, starting from the gating on single cells based on their bright field aspect ratio intensity and area. These single cells were restricted to those being in the focus of the camera, followed by distinguishing between non-infected and infected cells based on the intensity of the GFP signal. Finally, the mean and median fluorescence intensity resulting from the incorporated galactose derivative (Alexa-

Fluor-568) was analyzed and represented as a ratio to the corresponding control. The presented data result from as a pool from two individual experiments which were performed with technical duplicates.

3. NMR Spectra

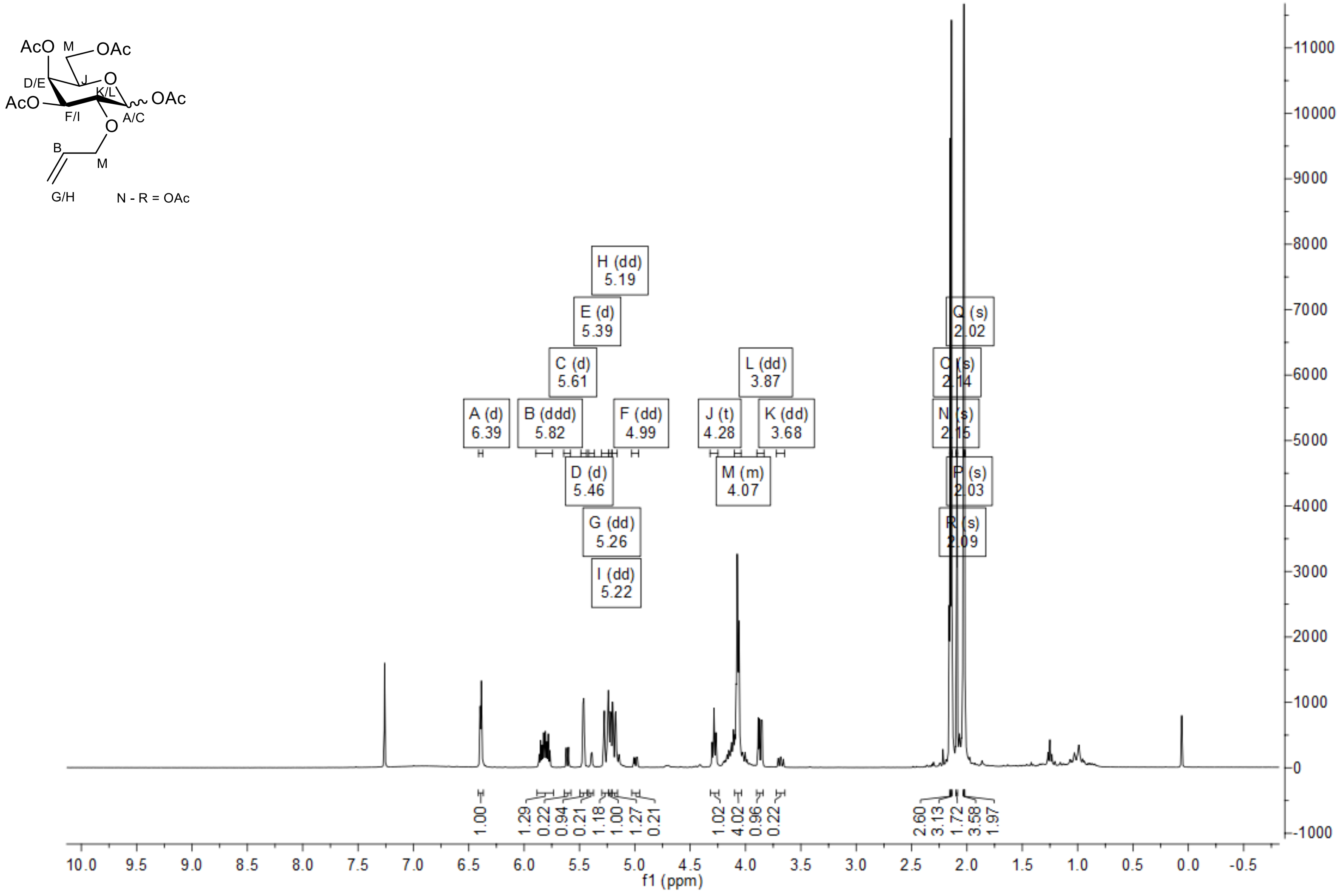

Figure S15: ^{1}H NMR of **2a** ($CDCl_3$, 400 MHz).

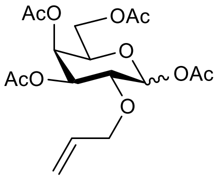

2a

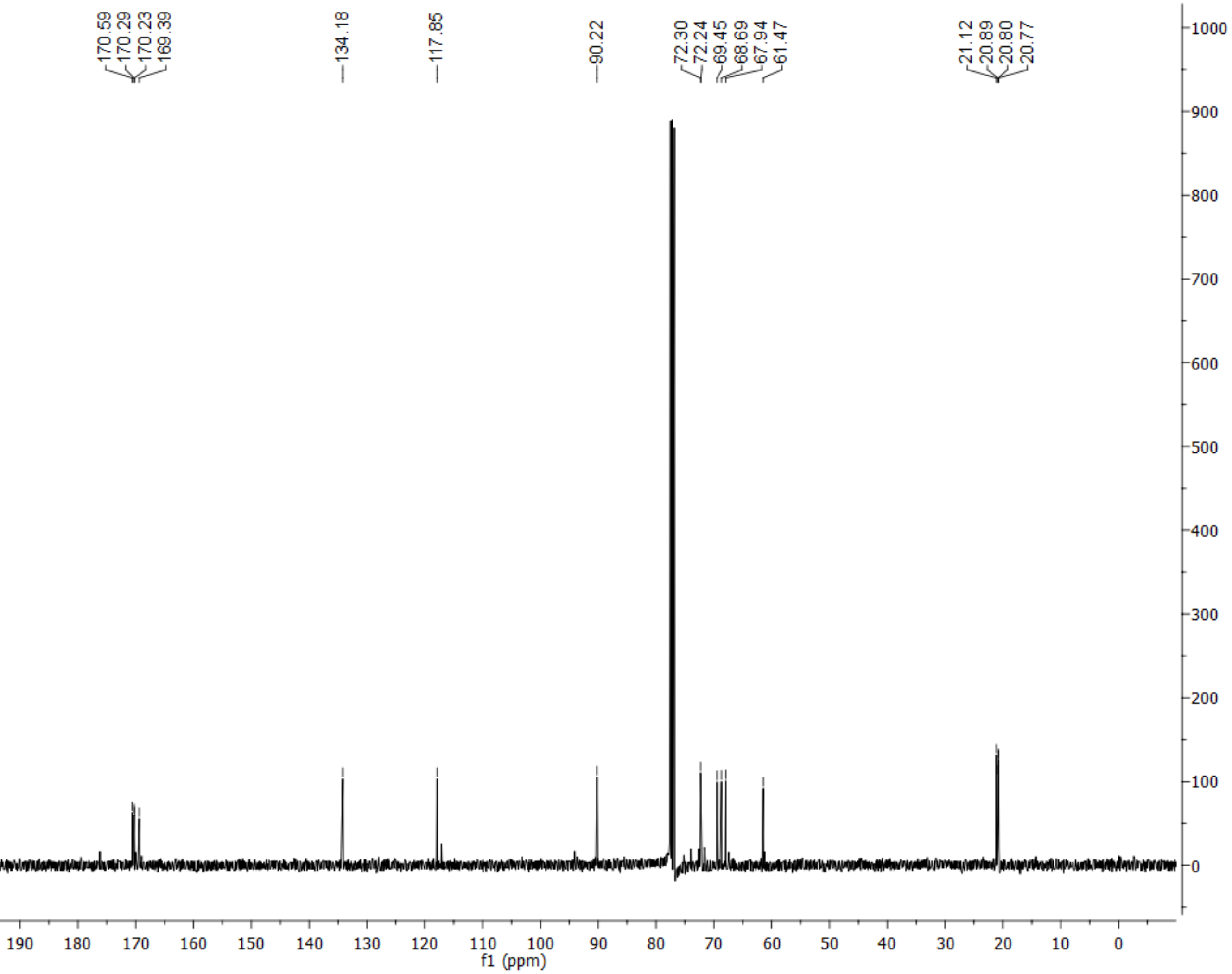

Figure S16: ^{13}C NMR of **2a** ($CDCl_3$, 100 MHz).

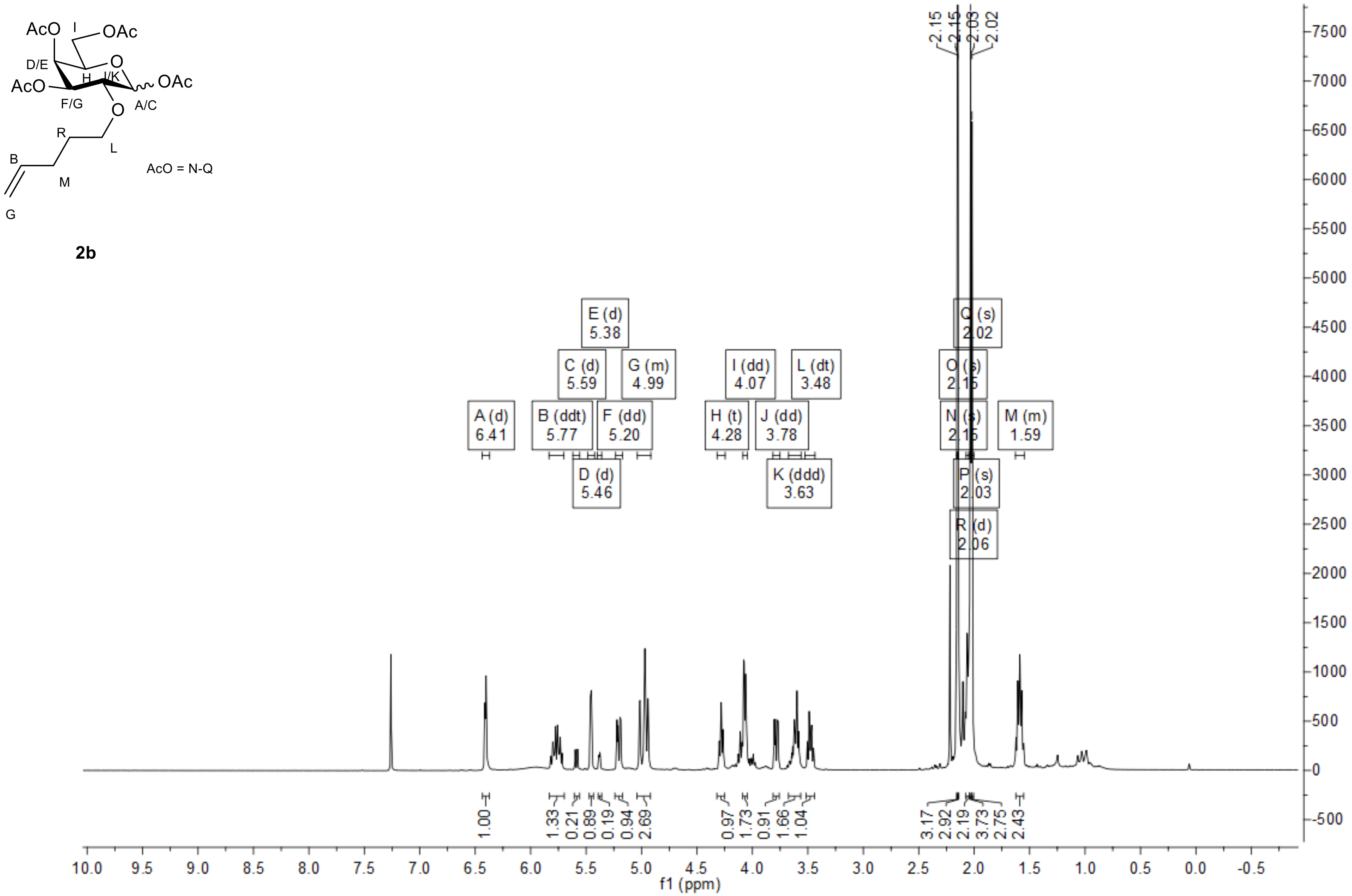

Figure S17: ^{1}H NMR of **2b** ($CDCl_3$, 400 MHz).

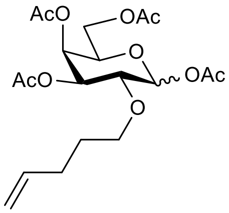

2b

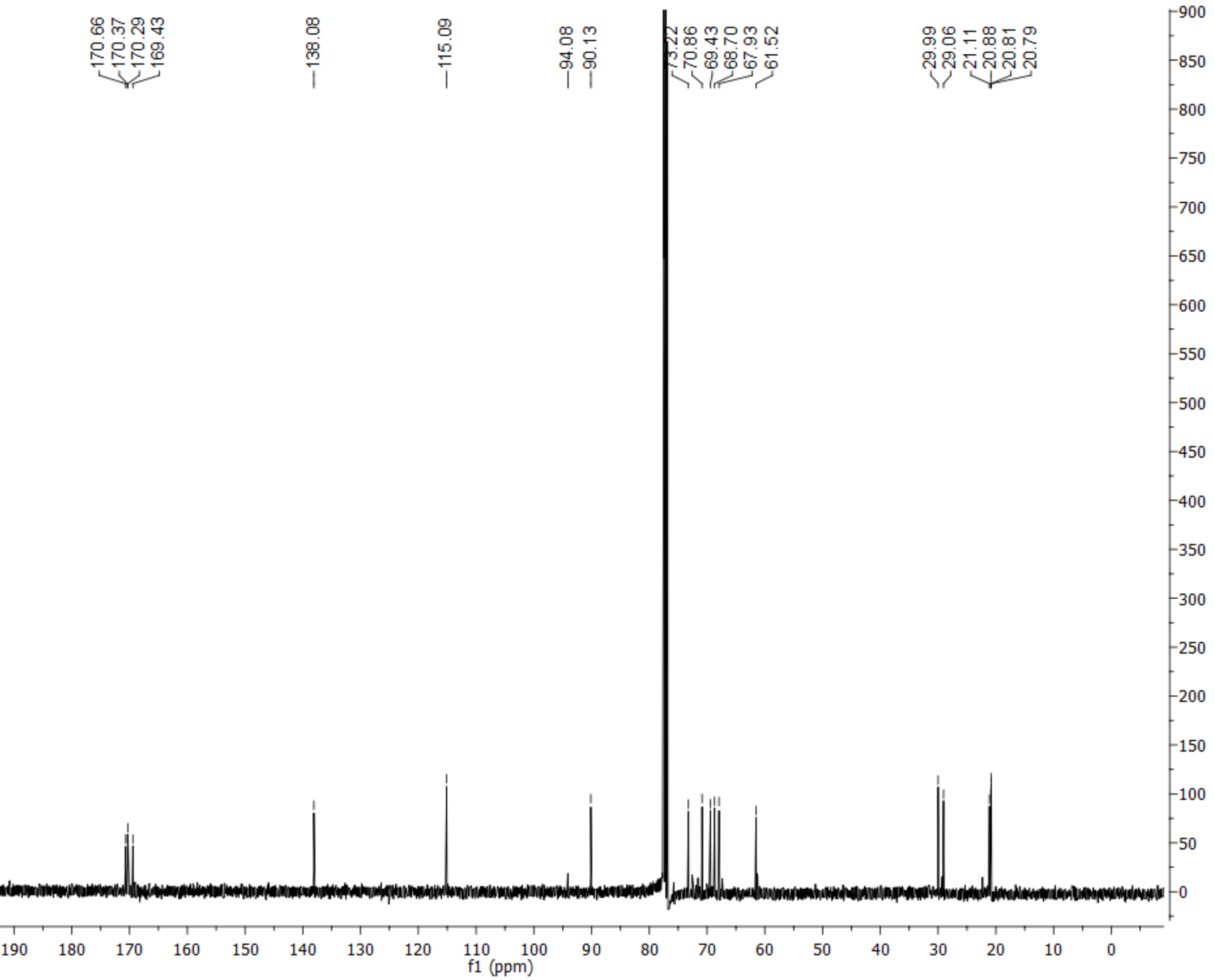

Figure S18: ^{13}C NMR of **2b** ($CDCl_3$, 100 MHz).

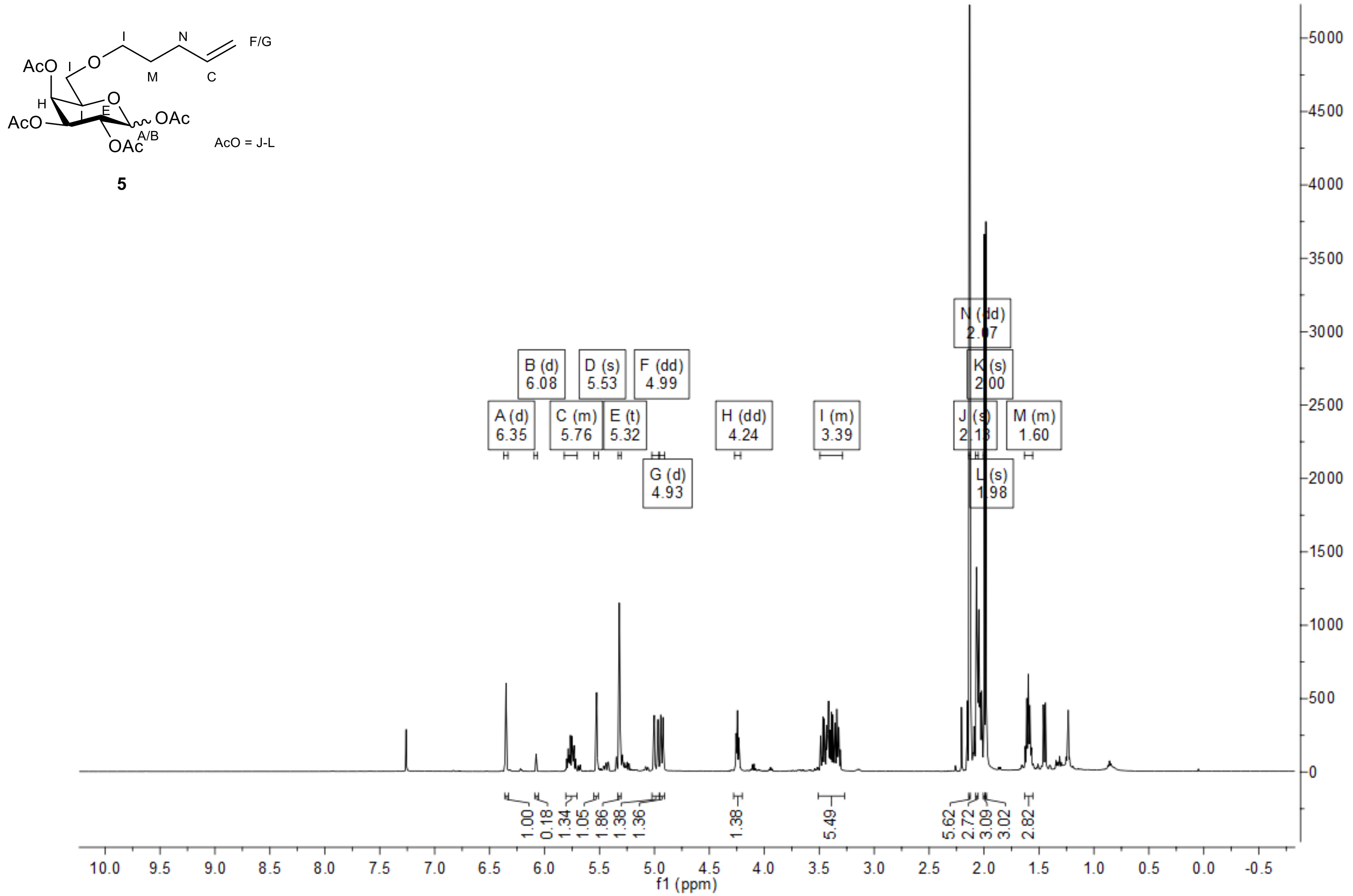

Figure S19: ^{1}H NMR of **5** ($CDCl_3$, 400 MHz).

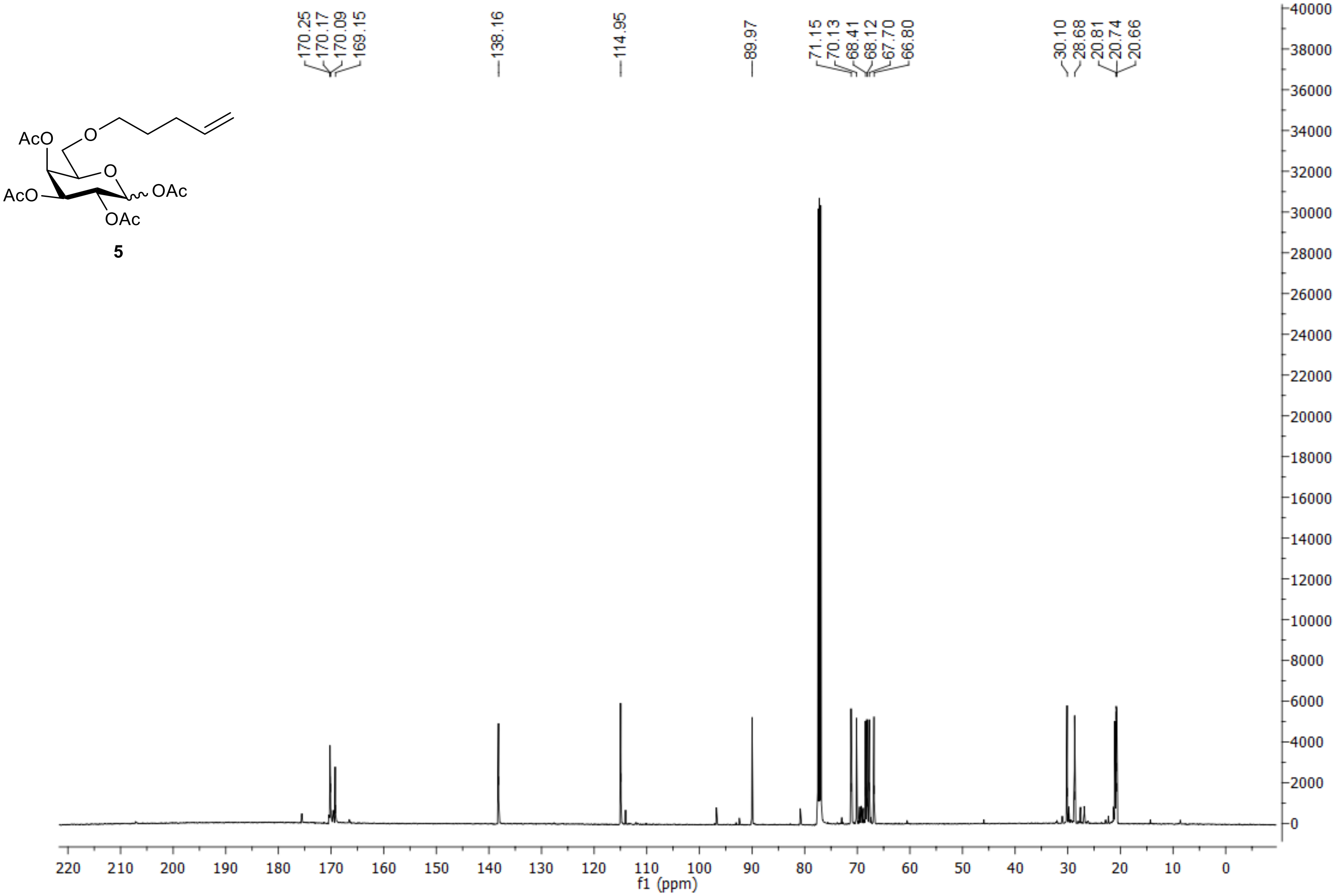

Figure S20: ^{13}C NMR of **5** ($CDCl_3$, 126 MHz).

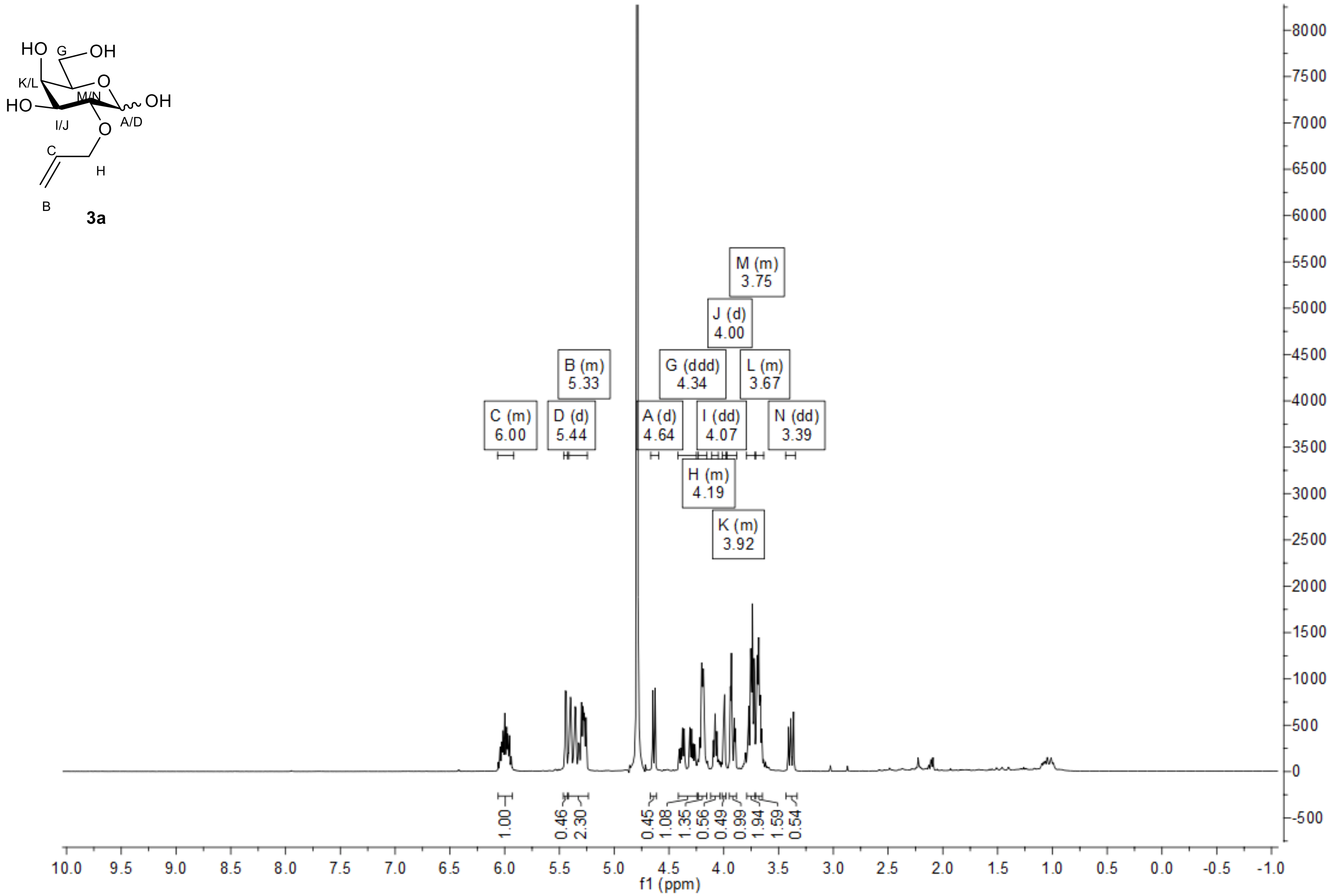

Figure S21: ^{1}H NMR of **3a** (D_2O, 400 MHz).

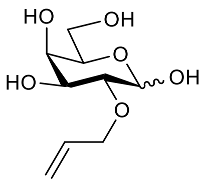

3a

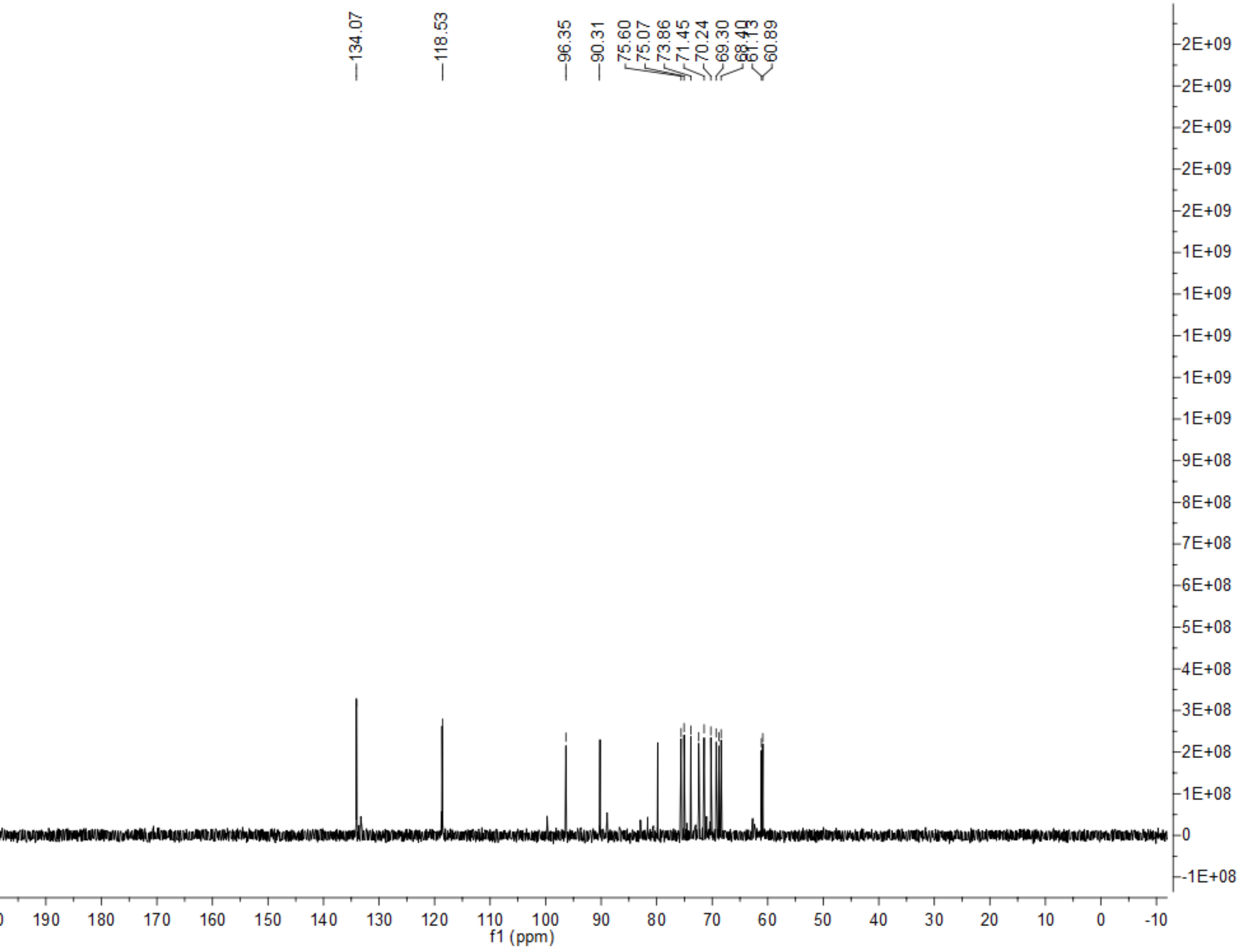

Figure S22: ^{13}C NMR of **3a** (D_2O, 100 MHz).

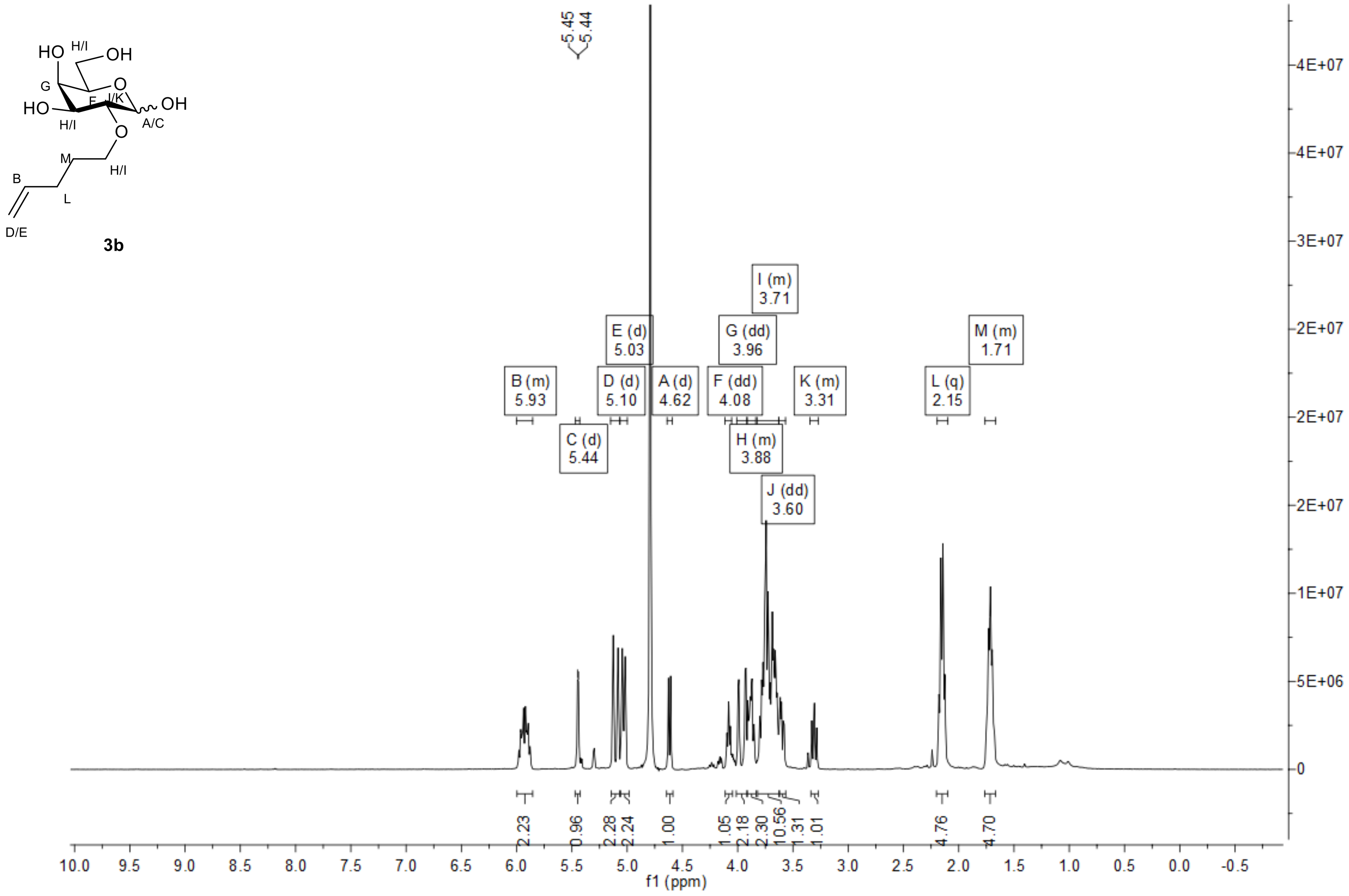

Figure S23: ^{1}H NMR of **3b** (D_2O, 400 MHz).

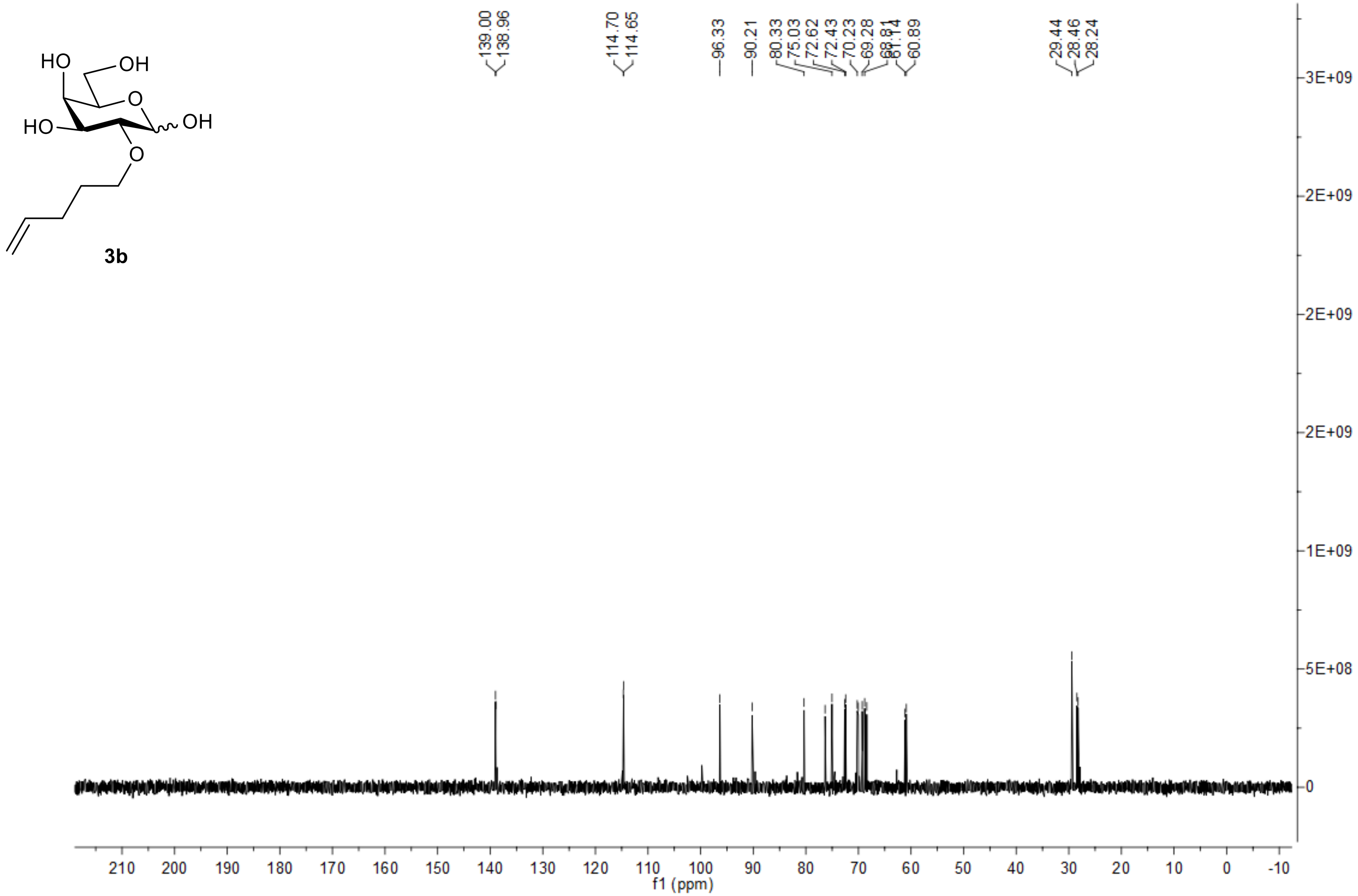

Figure S24: ^{13}C NMR of **3b** (D_2O, 100 MHz).

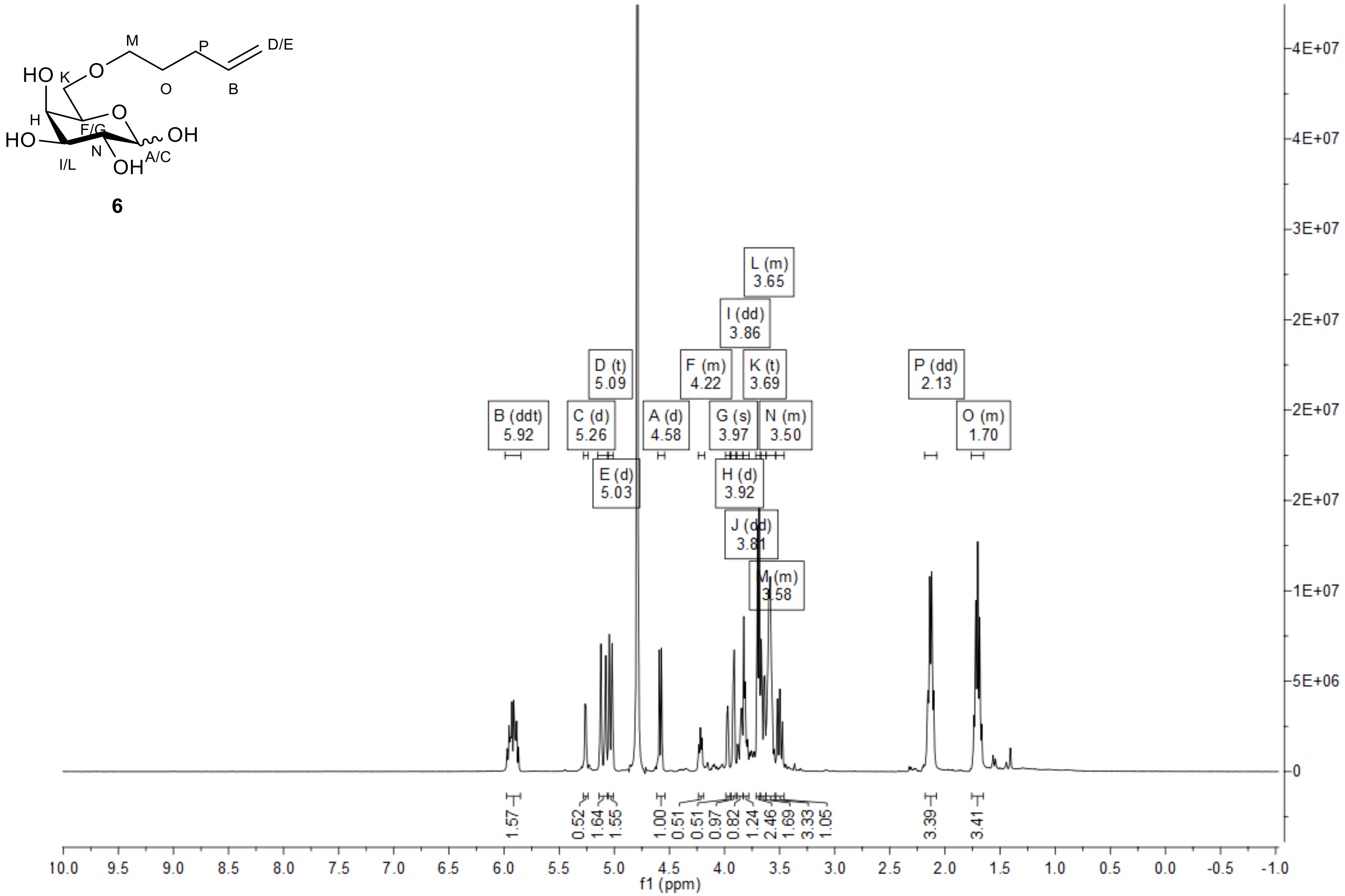

Figure S25: ^{1}H NMR of **6** (D_2O, 400 MHz).

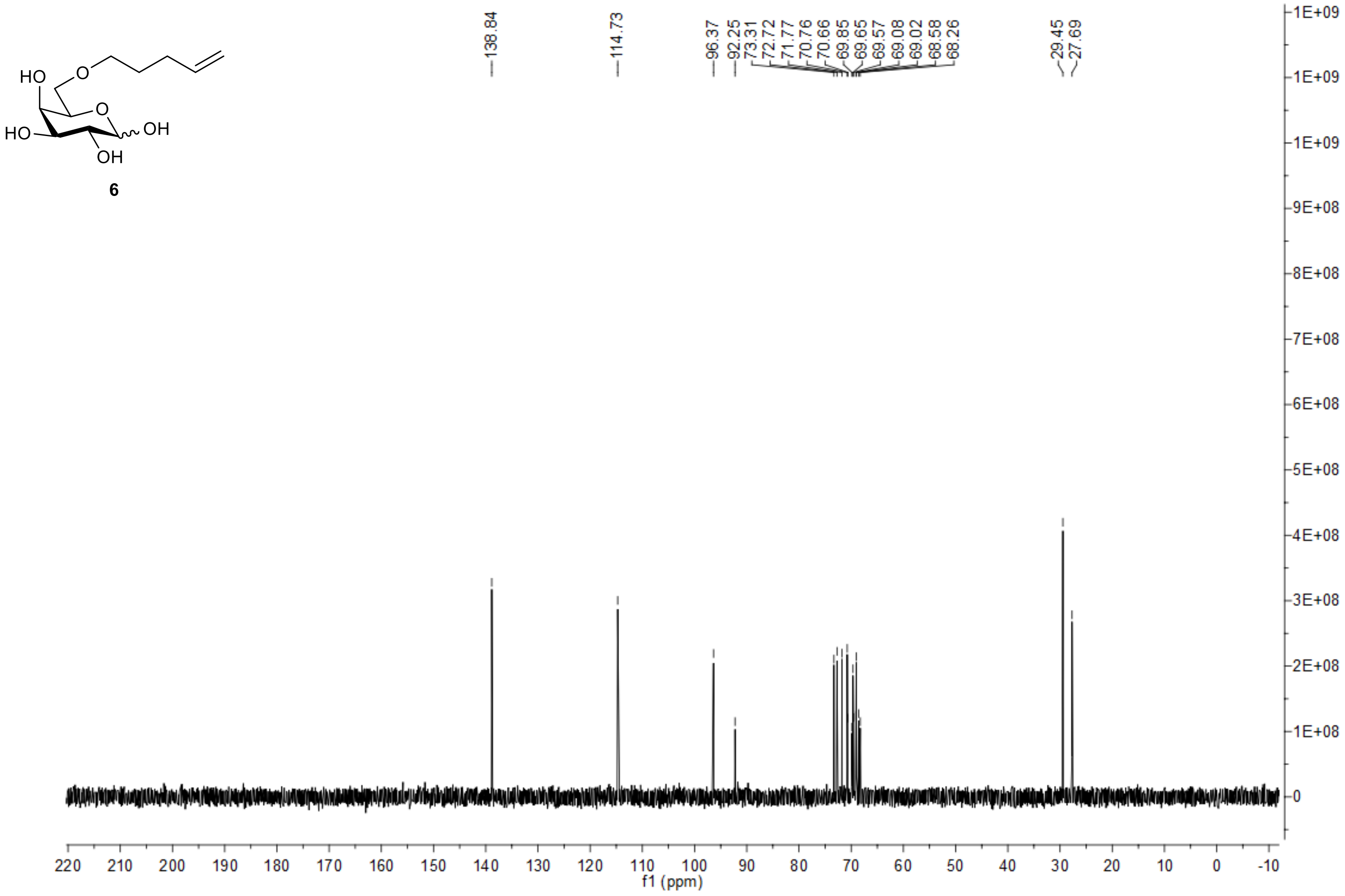

Figure S26: ^{13}C NMR of **6** (D_2O, 100 MHz).
